# Fine-Mapping and Comparative Genomic Analysis Reveal the Gene Composition at the *S* and *Z* Self-incompatibility Loci in Grasses

**DOI:** 10.1093/molbev/msac259

**Published:** 2022-12-08

**Authors:** Marius Rohner, Chloé Manzanares, Steven Yates, Daniel Thorogood, Dario Copetti, Thomas Lübberstedt, Torben Asp, Bruno Studer

**Affiliations:** Molecular Plant Breeding, Institute of Agricultural Sciences, ETH Zurich, Zurich, Switzerland; Molecular Plant Breeding, Institute of Agricultural Sciences, ETH Zurich, Zurich, Switzerland; Molecular Plant Breeding, Institute of Agricultural Sciences, ETH Zurich, Zurich, Switzerland; Institute of Biological, Environmental and Rural Sciences (IBERS), Aberystwyth University, Aberystwyth, United Kingdom; Molecular Plant Breeding, Institute of Agricultural Sciences, ETH Zurich, Zurich, Switzerland; Arizona Genomics Institute, School of Plant Sciences, College of Agriculture and Life Sciences, University of Arizona, Tucson, AZ, USA; Department of Agronomy, Iowa State University, Ames, IA, USA; Center for Quantitative Genetics and Genomics, Faculty of Technical Sciences, Aarhus University, Slagelse, Denmark; Molecular Plant Breeding, Institute of Agricultural Sciences, ETH Zurich, Zurich, Switzerland

**Keywords:** self-incompatibility (SI), Poaceae, perennial ryegrass (*Lolium perenne* L.), *SDUF247-I*, *SDUF247-II*, *ZDUF247-I*, *ZDUF247-II*, *DUF247*, *sS*, *sZ*

## Abstract

Self-incompatibility (SI) is a genetic mechanism of hermaphroditic plants to prevent inbreeding after self-pollination. Allogamous Poaceae species exhibit a unique gametophytic SI system controlled by two multi-allelic and independent loci, *S* and *Z*. Despite intense research efforts in the last decades, the genes that determine the initial recognition mechanism are yet to be identified. Here, we report the fine-mapping of the *Z*-locus in perennial ryegrass (*Lolium perenne* L.) and provide evidence that the pollen and stigma components are determined by two genes encoding DUF247 domain proteins (*ZDUF247-I* and *ZDUF247-II*) and the gene *sZ*, respectively. The pollen and stigma determinants are located side-by-side and were genetically linked in 10,245 individuals of two independent mapping populations segregating for *Z*. Moreover, they exhibited high allelic diversity as well as tissue-specific gene expression, matching the expected characteristics of SI determinants known from other systems. Revisiting the *S*-locus using the latest high-quality whole-genome assemblies revealed a similar gene composition and structure as found for *Z*, supporting the hypothesis of a duplicated origin of the two-locus SI system of grasses. Ultimately, comparative genomic analyses across a wide range of self-compatible and self-incompatible Poaceae species revealed that the absence of a functional copy of at least one of the six putative SI determinants is accompanied by a self-compatible phenotype. Our study provides new insights into the origin and evolution of the unique gametophytic SI system in one of the largest and economically most important plant families.

## Introduction

The mating systems and mechanisms behind sexual reproduction of flowering plants are diverse: monoecious plants produce flowers with only one reproductive organ, either female or male, promoting cross-pollination (Willson 1983). Hermaphroditic plants developed various strategies promoting cross-pollination, determined, for example, by the morphology of the reproductive organs (Ganders 1979) or by differences in the maturity of these organs (Lloyd and Webb 1986).

Self-incompatibility (SI) is a mechanism preventing self-pollination upon self-pollen recognition by the female organ. Different genetic mechanisms exist in angiosperms (De Nettancourt 1977; Takayama and Isogai 2005). In most flowering plants, the recognition of self-pollen by the pistil is genetically controlled by a single multi-allelic locus, the *S*-locus. The *S*-locus encodes at least two closely linked genes, representing the male and female SI determinants (Fujii et al. 2016). The same *S*-allele specificity expressed by the pollen and the pistil will halt pollen tube development and hence, successful fertilization (Takayama and Isogai 2005).

In-depth knowledge about the underlying genetic control has been acquired for three single locus multi-allelic SI systems: the *S*-RNase type SI system (McClure et al. 1989; Kao and Tsukamoto 2004; Sijacic et al. 2004; Williams et al. 2015; Sassa 2016), the Papaveraceae type SI system (Foote et al. 1994; Wheeler et al. 2009; Poulter et al. 2010; Wilkins et al. 2014; Wang et al. 2018), and the Brassicaceae type SI system (Nasrallah et al. 1987; Schopfer et al. 1999; Takasaki et al. 2000; Sehgal and Singh 2018). The diverse identity of the genetic determinants in these well-studied SI systems strongly supports the hypothesis that different SI systems have evolved independently in different lineages (Steinbachs and Holsinger 2002; Charlesworth et al. 2005). Despite their profound differences, several evolutionary features are shared, such as the high allelic and nucleotide diversity within a species but also the low nucleotide variation between the SI determinants of the same allelic specificity (Charlesworth et al. 2005). Furthermore, the suppression of recombination between the male and female SI determinants in SI systems is considered essential, as a recombination event may produce a non-functional SI haplotype leading to the breakdown of SI (Fujii et al. 2016).

Self-incompatibility in the grass family (Poaceae) is yet to be elucidated, despite early research by Lundqvist dating back to 1954 (Lundqvist 1954). In grasses, SI is reported in many tribes such as Triticeae (*Secale cereale* L., *Hordeum bulbosum* L.), Paniceae (*Panicum virgatum* L.), Oryzeae (*Oryza longistaminata* A. Chev. & Roehr), Andropogoneae (*Miscanthus sinensis* Anderss.), and the Poeae (*Festuca pratensis* Huds., *Lolium perenne* L., *Lolium multiflorum* Lam.) (see Li et al. (1997) and Do Canto et al. (2016) for a complete list). The SI system in grasses is gametophytically controlled and genetically governed by two multi-allelic and independent loci, *S* and *Z* (Lundqvist 1954; Hayman 1956; Cornish et al. 1979). Self-recognition is based on the interaction between male and female determinants of both loci. The fertilization is halted when *S*- and *Z*-haplotypes of the pollen are matched in the stigma. The recognition of self-incompatible pollen in grasses, followed by the inhibition of the pollen tube growth, is very rapid, occurring at the stigma surface within minutes after germination (Shivanna et al. 1982). The downstream reaction upon the initial self/nonself-recognition is unknown. The involvement of Ca^2+^-induced signaling transduction, protein phosphorylation, and the proteolysis pathway have been reported in preliminary studies (Wehling et al. 1994; Klaas et al. 2011). The current knowledge suggests that self-incompatible species of the entire Poaceae family share the same SI system, similarly as all dicotyledonous species investigated at the molecular level belonging to the same family share the same SI system (Li et al. 1997; Baumann et al. 2000). In perennial ryegrass (*L. perenne*), the *S*- and the *Z*-locus have been mapped to chromosomes 1 and 2, respectively, using genetic linkage mapping (Thorogood et al. 2002). These two loci have also been located on chromosomes 1 and 2 of rye (*S. cereale*; Wricke and Wehling 1985; Gertz and Wricke 1989) and sunolgrass (*Phalaris coerulescens* Desf.; Bian et al. 2004), for example. The syntenic region in self-compatible rice (*Oryza sativa* L.) can be found on chromosome 5 for *S* and chromosome 4 for *Z* (Jones et al. 2002).

More recently, the *S*-locus has been mapped to a 0.1 centimorgan (cM) region by Manzanares et al. (2016) in perennial ryegrass, containing eight genes. The gene *SDUF247* (or *LpSDUF247*, as isolated in *L. perenne*) has been suggested as the gene encoding for the pollen component, due to its high sequence diversity and the fact that the allelic sequences observed at that gene were fully predictive for the *S*-locus genotypes known to segregate in the population used for fine-mapping. Furthermore, within *SDUF247*, a frameshift mutation has been identified within self-compatible darnel (*Lolium temulentum* L.), whereas all self-incompatible species analyzed within the *Festuca-Lolium* species complex were predicted to encode functional SDUF247 proteins. However, due to the absence of a contiguous genome sequence at the *S*-locus, the identity of the female component remained elusive (Manzanares et al. 2016).

Fine-mapping of the *Z*-locus is less advanced: In rye, the genomic region containing the *Z*-locus was narrowed down to 1.5 cM on chromosome 2RL (Hackauf and Wehling 2005). Shinozuka et al. (2010) identified the orthologous region spanning 60 kb on chromosome 5 in *Brachypodium distachyon* (L.) P. Beauv. Using a comparative genomics approach based on the synteny between chromosome 5 of *B. distachyon* and chromosome 2 of perennial ryegrass, BAC clones co-segregating with the *Z*-locus were identified and used for sequencing. From this study, a gene encoding for a protein containing a DUF247 domain has been identified in the *Z*-locus region of perennial ryegrass, as well as three other candidate genes (Shinozuka et al. 2010).

Longstamen rice (*O. longistaminata*), a self-incompatible African rice species, was recently reported to have maintained the two-locus gametophytic SI system of grasses (Lian et al. 2021). Comparative genomic analysis enabled the identification of the gene orthologous to the putative male *S*-locus determinant of perennial ryegrass (*LpSDUF247*). The gene named *OlSS1* encodes for a member of the DUF247 protein family. A second gene (*OlSS2*), also predicted to encode for a protein of the DUF247 family, was identified nearby. Sequence polymorphism analysis of the genes adjacent to *OlSS* led to the identification of a possible female determinant at *S*, *OlSP*. The *OlSP* gene contains an N-terminal YfaZ domain of unknown function, and an ortholog to this gene in *H. bulbosum* (*HPS10*) has been previously presented as a possible candidate for the female determinant at the *S*-locus (Kakeda et al. 2008; Kakeda 2009). The reported high density of sequence polymorphisms and expression data for the identified genes at the *S*-locus in *O. longistaminata* showed that *OlSS1* and *OlSS2* are plausible candidate genes for the male determinant, whereas *OlSP* likely encodes for the female determinant at *S* (Lian et al. 2021).

Whole-genome sequences and high-quality assemblies thereof have been established for several major self-compatible crop species within the Poaceae family, for example for rice (*O. sativa*; Stein et al. 2018), maize (*Zea mays* L.; Schnable et al. 2009), barley (*Hordeum vulgare* L.; Mayer et al. 2012), rye (*S. cereale*; Li et al. 2021; Rabanus-Wallace et al. 2021), wheat (*Triticum aestivum* L.; Appels et al. 2018), and purple false brome (*B. distachyon*; Vogel et al. 2010). In contrast, the genomic resources available for outbreeding forage grasses like perennial ryegrass, Italian ryegrass (*L. multiflorum*), orchardgrass (*Dactylis glomerata* L.), and meadow fescue (*F. pratensis*) are limited. The primary limitations hampering the development of high-quality genome assemblies within outbreeding forage grasses are their high level of heterozygosity and the high content of repetitive sequences within the genome (Byrne et al. 2015). In recent years, more contiguous genome assemblies have become available for forage grasses and non-major crop species, including two reference-grade genome assemblies of perennial ryegrass (Frei et al. 2021; Nagy et al. 2022), a high-quality draft diploid genome assembly of Italian ryegrass (Copetti et al. 2021), and a chromosome-scale diploid genome assembly of orchardgrass (Huang et al. 2020). The concurrent availability of high-quality Poaceae genome assemblies from self-incompatible and self-compatible species finally allows for an intensive comparative genomics approach to investigate the underlying genetic basis of SI.

The main objective of this study was to further characterize and advance our understanding of the two-locus gametophytic SI system in Poaceae species by identifying the male and female determinants at the *S*- and *Z*-locus. Specifically, we aimed to locate the *Z*-locus through fine-mapping in perennial ryegrass using a number of mapping individuals sufficiently high to reach gene-scale resolution. Learning from the gene composition, order, and orientation at the *Z*-locus, we further aimed to reconstruct the gene content at the *S*-locus and compare it to other species of the *Festuca-Lolium* species complex and grasses in general. Finally, through a complementary set of genetic analyses, including sequence diversity and gene expression analysis, we aimed to identify the *S*- and *Z*-locus determinants and distinguish between the male and female components of the SI system present in the family of grasses.

## Results

### Fine-mapping of the *Z*-locus in Perennial Ryegrass

A total of 10,245 plants from two genetically unrelated perennial ryegrass populations, hereafter referred to as VrnA-XL and DTZ, were used for fine-mapping. With a similar approach as described by Manzanares et al. (2016), the two markers CADELP and Lp02_555 flanking the *Z*-locus identified a total of 89 and 99 recombination events in VrnA-XL and DTZ, respectively (supplementary table S1, Supplementary Material online).

**Fig. 1.**
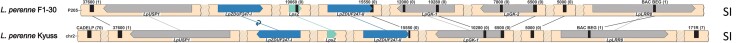
The gene composition of the genome region co-segregating with the *Z*-locus in perennial ryegrass (*Lolium perenne* L.). Given are the genotypes F1-30 (haplotype P205, above) and the doubled haploid genotype Kyuss (below). The sequence of the *Z*-locus is continuous, but for clarity, the gene- and marker-less regions are represented as shaded breaks. The genes are represented with bars, and their orientation is shown with the pointy side representing the 3′ end. The self-incompatibility candidate genes are colored in teal and blue. The markers used for the fine-mapping are represented by black bars, and the number of recombinants for each marker is indicated between brackets. The synteny between homologous genes is illustrated with lines connecting the two haplotypes, and in case of orientation change, a small circular arrow is used. On the right, the compatibility phenotype is indicated (SI, self-incompatible).

To establish the DNA sequence at the *Z*-locus and to locate the recombination events, the perennial ryegrass BAC libraries described by Farrar et al. (2007) were screened using the marker TC116908 (Hackauf and Wehling 2005). The BAC library constructed from the genotype NV#20F1-30 (hereafter referred to as F1-30) was particularly suitable, as F1-30 is one of the two parental genotypes that was used to develop VrnA-XL. The BAC clone, identified to contain the *Z*-locus of F1-30, was grown, its DNA was isolated, and sequenced. Sequence assembly reconstructed a 99,618 bp long single contig of P205C9H17P (GenBank accession number OP292309), which was used to develop DNA markers for fine-mapping (supplementary table S1, Supplementary Material online). Projection of the recombination events from the two different fine-mapping populations VrnA-XL and DTZ on P205C9H17P identified a region of 37,125 bp co-segregating with the *Z*-locus (hereafter referred to as haplotype P205), delimited by the markers BAC_BEG and 37600 (supplementary table S1, Supplementary Material online and fig. 1).

The annotation of the genome region co-segregating with the *Z*-locus was done using available genomic resources (Byrne et al. 2015; Begheyn et al. 2018; Copetti et al. 2021; Frei et al. 2021; Nagy et al. 2022), gene prediction software (Stanke and Morgenstern 2005), and a manual BLAST-based approach. Six genes were identified on P205: *LpUSP1*, *LpZDUF247-I*, *Lolium perenne* stigma *Z* (*LpsZ*), *LpZDUF247-II*, *LpGK*, and *LpLRR8* (fig. 1 and table 1). The *Z*-locus as revealed for P205 was compared with the reference-grade perennial ryegrass genome of the doubled haploid genotype Kyuss (Frei et al. 2021): While the overall gene order was conserved between the two perennial ryegrass haplotypes, the partial duplication of *LpGK* on P205 (leading to *LpGK-1* and *LpGK-2*) was missing in Kyuss. Furthermore, the orientation of *LpZDUF247-I* was not conserved between the two perennial ryegrass genotypes (fig. 1). Two *Z*-locus genes containing a DUF247 domain were present in both genotypes and were annotated as two different genes (*LpZDUF247-I* and *LpZDUF247-II*), their nucleotide sequence being too different to be considered as a recent gene duplication.

**Table 1.** Gene Composition at the *Z*- and the *S*-Locus of the Gametophytic Self-incompatibility (SI) System in Ryegrass (*Lolium* spp.).

SI locus	Gene name	Gene annotation name			Gene description NCBI
		*Lolium perenne* P226/135/16 (inbred)^a^	*Lolium perenne* Kyuss (doubled haploid)^b^	*Lolium multiflorum* Rabiosa (heterozygous)^c^	
*Z*	*USP1*	*XLOC_023214*	*KYUS_G_chr2.53349*	*Lmu01_1905G0001430* & *Lmu01_3448G0000660*	Ubiquitin carboxyl-terminal hydrolase
*Z*	*ZDUF247-I*	*XLOC_014562*	*KYUS_G_chr2.53348*	*Lmu01_1905G0001490* & *Lmu01_3448G0000640*	DUF247; Plant protein of unknown function
*Z*	*sZ*	*XLOC_023217*	*KYUS_G_chr2.53336*	*Lmu01_1905G0001500* & *Lmu01_3448G0000650*	Conserved hypothetical protein
*Z*	*ZDUF247-II*	*XLOC_014562*	chr2 14505344...14507005^d^	*Lmu01_1905G0001500* & scf3448 1166016...1167647^d^	DUF247; Plant protein of unknown function
*Z*	*GK-1*	*XLOC_008351*	*KYUS_G_chr2.53334*	*Lmu01_1905G0001510* & *Lmu01_3448G0000620*	Glycerol kinase
*Z*	*GK-2*	*XLOC_014564*	NA^e^	NA^e^	Glycerol kinase
*Z*	*LRR8*	*XLOC_008352*	*KYUS_G_chr2.53330*	*Lmu01_1905G0001520* & *Lmu01_3448G0000600*	LRR receptor-like protein
*S*	*RecQ*	*XLOC_040775*	*KYUS_G_chr1.6323*	*Lmu01_818G0000120* & *Lmu01_1212G0000470*	Putative DNA helicase RecQ
*S*	*TIR1*	*XLOC_005302*	*KYUS_G_chr1.6316*	*Lmu01_818G0000130* & *Lmu01_1212G0000460*	Transport inhibitor response 1-like protein
*S*	*dsRNAbp*	*XLOC_005304*	*KYUS_G_chr1.6309*	*Lmu01_818G0000140* & *Lmu01_1212G0000450*	Double-stranded RNA-binding protein 2
*S*	*SNF2*	*XLOC_005306*	*KYUS_G_chr1.6308*	*Lmu01_818G0000150* & *Lmu01_1212G0000440*	Probable chromatin-remodeling complex ATPase chain
*S*	*SDUF247-I*	chr1 45765361...45766956^d^	chr1 224982167...224983759^d^	*Lmu01_818G0000190* & *Lmu01_1212G0000400*	DUF247; Plant protein of unknown function
*S*	*sS*	*XLOC_013962*	chr1 224985003...224984683^d^	*Lmu01_818G0000200* & *Lmu01_1212G0000430*	Conserved hypothetical protein
*S*	*SDUF247-II*	chr 1 45834579...45836240^d^	*KYUS_G_chr1.6214*	*Lmu01_818G0000210* & scf1212 4724980...4726635^d^	DUF247; Plant protein of unknown function
*S*	*PLP-2*	*XLOC_040815*	NA^e^	NA^e^ & *Lmu01_1212G0000360*	Pyridoxal phosphate homeostasis protein
*S*	*NBS-LRR-2*	*XLOC_040814*	NA^e^	NA^e^ & *Lmu01_1212G0000350*	NBS-LRR-like resistance protein
*S*	*PLP-1*	*XLOC_040815*	*KYUS_G_chr1.6210*	*Lmu01_818G0000230* & *Lmu01_1212G0000340*	Pyridoxal phosphate homeostasis protein
*S*	*NBS-LRR-1*	*XLOC_040817*	*KYUS_G_chr1.6209*	*Lmu01_818G0000240* & *Lmu01_1212G0000330*	NBS-LRR-like resistance protein
*S*	*TPR-2*	NA^e^	NA^e^	*Lmu01_818G0000280* & NA^e^	Anaphase-promoting complex subunit 7
*S*	*TPR-1*	*XLOC_019013*	*KYUS_G_chr1.6161*	*Lmu01_818G0000290* & *Lmu01_1212G0000280*	Anaphase-promoting complex subunit 7
*S*	*Ca^2+^bp*	*XLOC_000861*	*KYUS_G_chr1.6125*	*Lmu01_818G0000300* & *Lmu01_1212G0000270*	Serine-protein phosphatase 2A regulatory subunit B

*Note*: The annotation is given for each gene of the three genotypes P226/135/16 (*Lolium perenne* L., inbred), Kyuss (*L. perenne*, doubled haploid), and the genotype M.02402/16 of the cultivar ‘Rabiosa’ (*Lolium multiflorum* Lam., heterozygous). Each gene is presented with a description of the function derived from the gene ortholog in the genus *Oryza* (NCBI, taxid 4527). Gene duplications are marked with an “–” plus an Arabic number at the end of the gene name.

a Genome sequence from Nagy et al. (2022) and annotation file from Begheyn et al. (2018).

b Genome sequence and annotation file from Frei et al. (2021).

c Genome sequence and annotation file from Copetti et al. (2021).

d Positions within a scaffold (scf) or chromosome (chr) are given as no annotation is present.

e No orthologous gene sequence was identified.

### Comparative Genomics: Synteny of the *S*- and the *Z*-locus in the Poeae Tribe and the Poaceae Family

The *S*-locus in perennial ryegrass, as described by Manzanares et al. (2016), contained six unique genes, and the putative male determinant was identified as a gene harboring a DUF247 domain (*LpSDUF247*, hereafter referred to as *LpSDUF247-I*). In order to establish a contiguous genome sequence covering the *S*-locus, thereby identifying genes potentially missed in the fragmented assembly used by Manzanares et al. (2016), a comparative genomics analysis with the available genome sequence resources of *Lolium* spp. (table 2) was applied. By such analysis, three additional genes were found: *LpTPR*, another gene encoding for a DUF247 domain-containing protein (*LpSDUF247-II*), and *Lolium perenne* stigma *S* (*LpsS*) (table 1).

**Table 2.** Genome Sequence Data Used for the Comparative Genome Analysis.

Species	Source	GenBank accession number	Accessed
*Lolium perenne* Kyuss	Frei et al. (2021)	GCA_019359855	July 2021
*Lolium perenne* P226/135/16	Byrne et al. (2015) ^a^ and Nagy et al. (2022)^a^	NA	July 2022
	Begheyn et al. (2018) ^b^		
*Lolium perenne* S23 Z	NA	OP292310-OP292318	NA
*Lolium multiflorum*	Copetti et al. (2021)	NA	July 2020
*Dactylis glomerata*	Huang et al. (2020)	GCA_007115705	June 2019
*Brachypodium distachyon*	Vogel et al. (2010)	GCA_000005505	June 2020
*Triticum aestivum*	Appels et al. (2018)	GCA_900519105	April 2020
*Hordeum vulgare*	Mayer et al. (2012)	GCA_901482405	June 2020
*Secale cereale* Lo7	Rabanus-Wallace et al. (2021)	GCA_900002355	March 2021
*Secale cereale* Weining	Li et al. (2021)	GCA_016097815	May 2021
*Leersia perrieri*	Stein et al. (2018)	GCA_000325765	June 2020
*Oryza sativa* subsp. *japonica*	Stein et al. (2018)	GCA_001433935	June 2020
*Oryza longistaminata*	Stein et al. (2018)	GCA_000789195	June 2020
*Setaria italica*	Bennetzen et al. (2012)	GCA_000263155	August 2020
*Zea mays*	Schnable et al. (2009)	GCA_902167145	February 2020
*Sorghum bicolor*	Paterson et al. (2009)	GCA_000003195	June 2020

*Note*: For each genome assembly used, the scientific paper describing it, the GenBank accession number, if available, and the access date are given.

a Articles describing the genome assembly.

b Article describing the genome annotation file used in this study.

Comparison of the structure and the annotation of the genes found at the *S*-locus with the six newly identified genes at the *Z*-locus revealed similarities between the two SI loci in *Lolium* spp. (table 1). The *SI-DUF247* genes (*SDUF247-I*, *SDUF247-II*, *ZDUF247-I*, and *ZDUF247-II*), as well as *sS* and *sZ*, are here referred to as SI candidates, based on the already identified male determinant (*SDUF247-I*) by Manzanares et al. (2016) and the potential duplicative origin of the two-locus SI system in grasses (Lundqvist 1962).

**Fig. 2.**
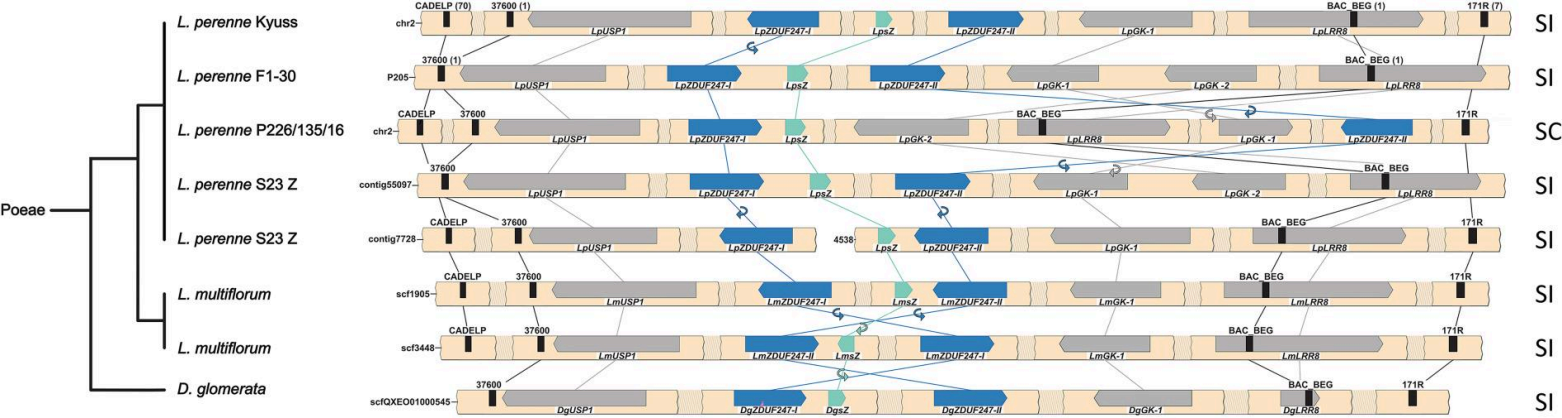
Synteny maps of the *Z*-locus of multiple genotypes from the Poeae tribe. For the *Lolium perenne* L. genotype S23 Z and *Lolium multiflorum* Lam., both haplotypes of the diploid assemblies are given. The phylogenetic tree (left), representing the different species, was drawn according to the NCBI taxonomy database. The gene- and marker-less regions are represented as shaded breaks, and a white space indicates an assembly gap. The genes present at the *Z*-locus are represented with directed arrows, and self-incompatibility candidate genes are colored in teal and blue. The gene orientation is shown with the pointy side representing the 3′ end. The markers used for the fine-mapping are represented by black bars, and the number of recombinants for each marker is indicated between brackets for *L. perenne* Kyuss and F1-30. The synteny between genes is illustrated by lines, and in case of orientation change, a small circular arrow is used. In addition, the compatibility phenotype of the genotype is indicated on the right: self-incompatible (SI) or self-compatible (SC).

**Fig. 3.**
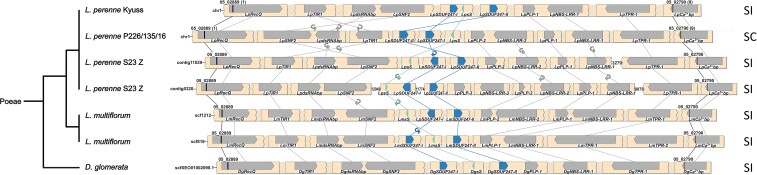
Synteny maps of the *S*-locus of multiple genotypes from the Poeae tribe. For the *Lolium perenne* L. genotype S23 Z and *Lolium multiflorum* Lam., both haplotypes of the diploid assemblies are given. The phylogenetic tree (left), representing the different species, was drawn according to the NCBI taxonomy database. The gene- and marker-less regions are represented as shaded breaks, and a white space indicates an assembly gap. The genes present at the *S*-locus are represented with directed arrows, and the self-incompatibility candidate genes are colored in teal and blue. The gene orientation is shown with the pointy side representing the 3′ end. The markers used for the fine-mapping are represented by black bars, and the number of recombinants for each marker is indicated between brackets for *L. perenne* Kyuss and P226/135/16. The synteny between genes is illustrated by lines, and in case of orientation change, a small circular arrow is used. In addition, the compatibility phenotype of the genotype is indicated on the right: self-incompatible (SI) or self-compatible (SC).

**Fig. 4.**
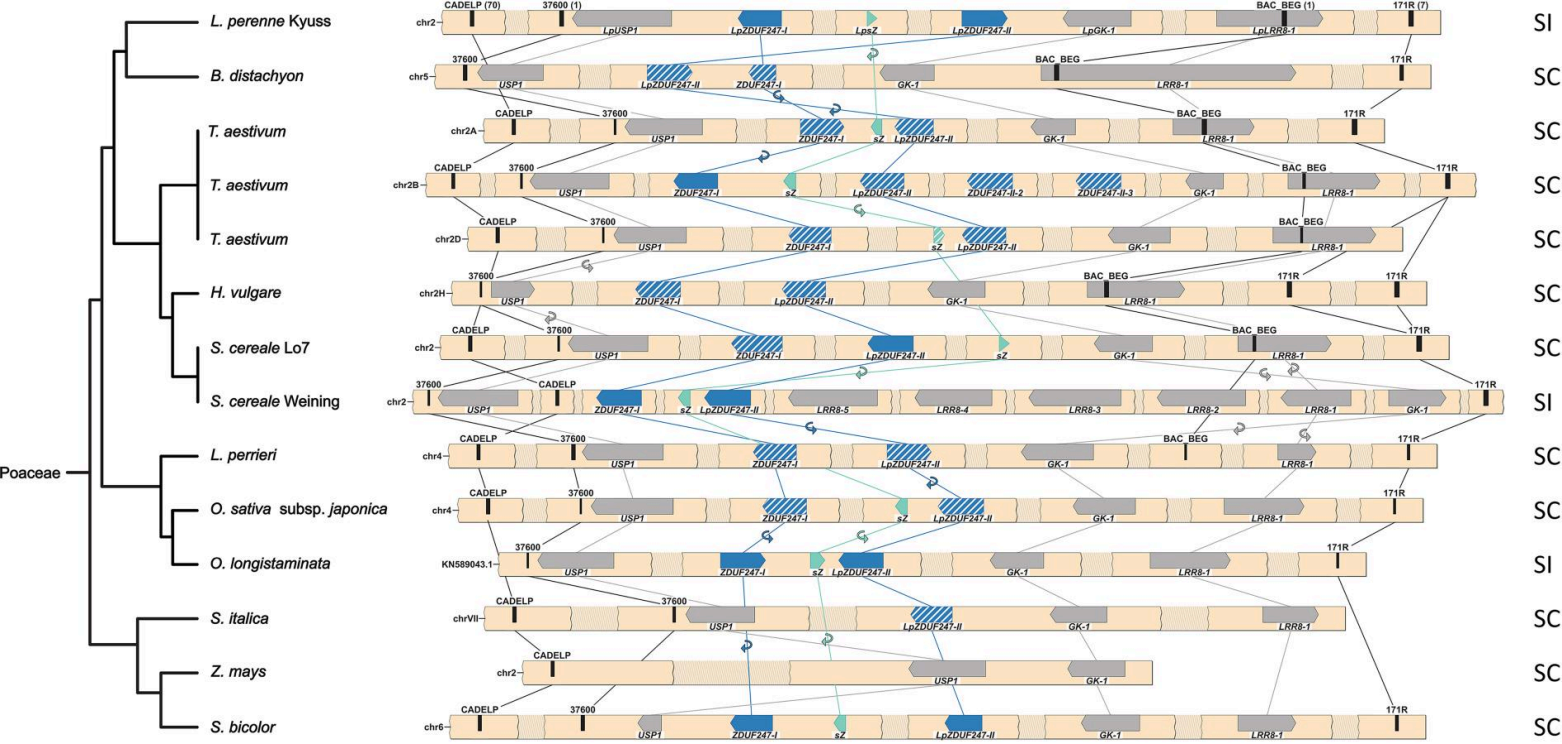
Synteny maps of the *Z*-locus of 11 Poaceae species. The phylogenetic tree (left), representing the different species, was drawn according to the NCBI taxonomy database. For allohexaploid *Triticum aestivum* L., the three homologous genomes A, B, and D are given. The gene- and marker-less regions are represented as shaded breaks, and a white space indicates an assembly gap. The genes present at the *Z*-locus are represented with directed arrows, and the self-incompatibility candidate genes are colored in teal and blue. A non-functional gene copy of the self-incompatibility candidates is indicated with a white striped pattern. The gene orientation is shown with the pointy side representing the 3′ end. The markers used for the fine-mapping are represented by black bars, and the number of recombinants for each marker is indicated between brackets for *L. perenne* Kyuss. The synteny between genes is illustrated by lines, and in case of orientation change, a small circular arrow is used. In addition, the compatibility phenotype is indicated on the right: self-incompatible (SI) or self-compatible (SC).

**Fig. 5.**
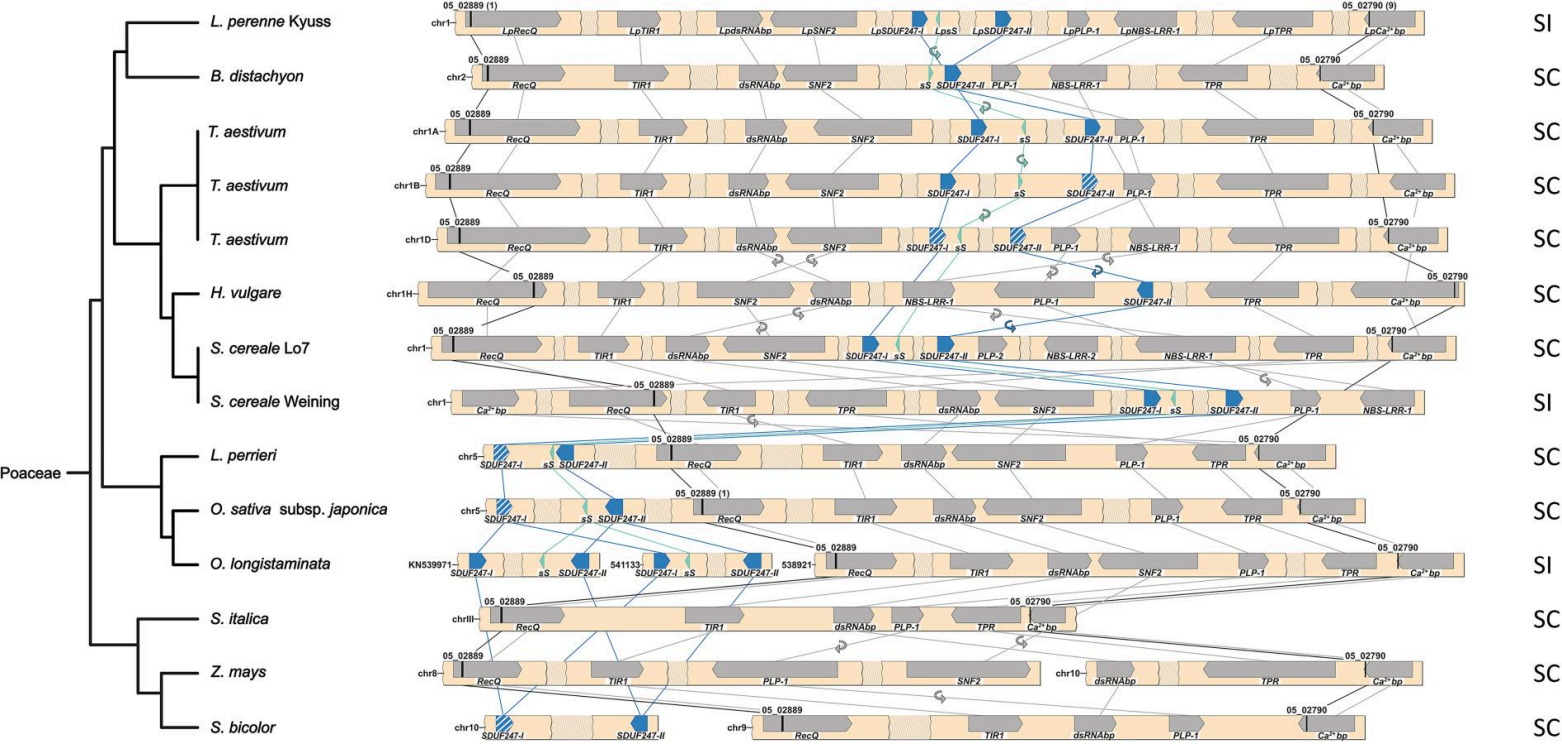
Synteny maps of the *S*-locus of 11 Poaceae species. The phylogenetic tree (left), representing the different species, was drawn according to the NCBI taxonomy database. For allohexaploid *Triticum aestivum* L., the three homologous genomes A, B, and D are given. The gene- and marker-less regions are represented as shaded breaks, and a white space indicates an assembly gap. The genes present at the *S*-locus are represented with directed arrows, and the self-incompatibility candidate genes are colored in teal and blue. A non-functional gene copy of the self-incompatibility candidates is indicated with a white striped pattern. The gene orientation is shown with the pointy side representing the 3′ end. The markers used for the fine-mapping are represented by black bars, and the number of recombinants for each marker is indicated between brackets for *L. perenne* Kyuss. Lines illustrate the synteny between genes, and in case of orientation change, a small circular arrow is used. In addition, the compatibility phenotype is indicated on the right: self-incompatible (SI) or self-compatible (SC).

To further study the gene content, order, and orientation at the *S*- and the *Z*-locus, the comparative genomic analysis was extended to include a wide range of self-compatible and self-incompatible species belonging to the tribe Poeae (figs. 2 and 3) and to the family Poaceae (figs. 4 and 5), as summarized in table 2.

Generally, a high level of genome synteny was observed at the *S*- and the *Z*-locus. The highest degree of synteny was found within species and genotypes from the Poeae tribe. Minor deviations included changes in the gene orientation, the duplication level of certain genes, and the gene order of the SI candidate genes (figs. 2 and 3). In contradiction to the high degree of synteny within the Poeae tribe stands the reference-grade genome assembly of the self-compatible *L. perenne* genotype P226/135/16, which displayed a unique gene order at both the *S*- and the *Z*-locus: At the *Z*-locus, the region harboring *LpGK-1* and *LpZDUF247-II* was inverted and reintegrated (fig. 2). At the *S*-locus, the region harboring *LpSNF2*, *LpdsRNAbp*, and *LpTIR* was also inverted (fig. 3).

The *S*- and the *Z*-locus in genotypes outside the Poeae tribe showed mainly a high synteny with the *S*- and the *Z*-locus of *Lolium* spp. (figs. 4 and 5), especially closely related species of the Triticaceae tribe (*T. aestivum*, *H. vulgare*, and *S. cereale*). Notable gene order alterations within the Triticaceae tribe were found in the *S. cereale* genotype Weining at the *S*-locus (fig. 5). A lower but comparable degree of synteny was observed within the Oryzeae tribe (*L. perrieri*, *O. longistaminata*, and *O. sativa* subsp. *japonica*) and *Sorghum bicolor* L., except that the SI candidate genes are located outside of the perennial ryegrass *S*-locus. The gene cluster consisting of *SDUF247-I*, *SDUF247-II*, and *sS* was not flanked by the perennial ryegrass flanking markers or the flanking genes. For *O. sativa* subsp. *japonica*, the SI candidate genes were 3.14 Mbp upstream of the flanking marker 05_02889 (Manzanares et al. 2016). In *L. perrieri*, the distance was 2.1 Mbp between the flanking marker 05_02889 and the SI candidate genes. For *O. longistaminata*, the SI candidate genes at *S* were present as duplication on individual scaffolds. However, whether the two copies result from a duplication or if both *S*-haplotypes were included in the haploid assembly remains elusive. In *S. bicolor*, the SI candidate genes were located on chromosome 10, whereas the *S*-locus flanking markers and flanking genes were localized on chromosome 8. In *S. italica* and *Z. mays*, almost no synteny could be observed at both loci, mainly through the absence of the SI candidate genes (figs. 4 and 5).

The functionality of the SI candidate genes and their orthologs was evaluated in addition to the synteny within the Poeae tribe and Poaceae family. A total of six *SDUF247* and seven *ZDUF247* gene pairs could be extracted from four perennial ryegrass genotypes (Kyuss, F1-30, P226/135/16, and S23 Z) and one Italian ryegrass genotype (genotype M.02402/16 of the cultivar ’Rabiosa’, hereafter referred to as genotype Rabiosa). All the extracted *SI-DUF247* genes shared the following characteristics: an intronless open reading frame (ORF) leading to a protein size of 508 to 559 amino acids (AAs), the translated protein belongs to the protein family DUF247 (pfam03140) and has a predicted non-cytoplasmic domain at the C-terminus, followed by a transmembrane domain and a small cytoplasmic domain at the N-terminus according to InterProScan. The six *sS* and seven *sZ* genes extracted from the same genotypes all shared the following characteristics: an ORF with one intron leading to a protein size of 82 to 122 AAs and a predicted signal peptide at the C-terminus, followed by a non-cytoplasmic domain according to InterProScan. These characteristics were used to assess the functionality of the SI candidate genes across the Poaceae family, that is, a gene was considered functional if all of the above-mentioned characteristics were met. Therefore, the assessment of the functionality of SI candidate genes is solely based on the genomic sequence; neither their expression nor their translation was taken into account.

**Fig. 6.**
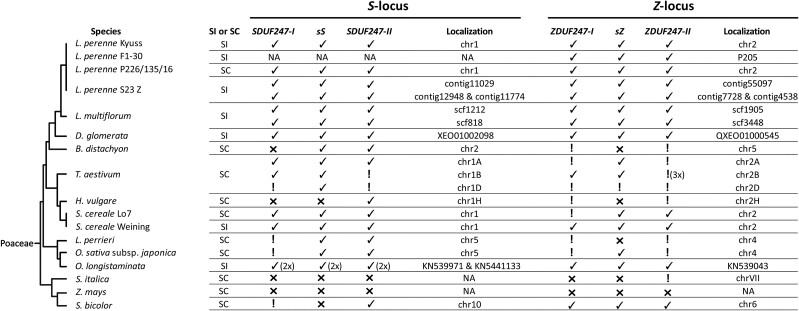
Composition of the self-incompatibility candidate genes in 17 genotypes representing 13 different Poaceae species. The phylogenetic tree (left), representing the different species, was drawn according to the NCBI taxonomy database. The compatibility phenotypes are indicated for each genotype: self-incompatible (SI) or self-compatible (SC). A checkmark (✓) represents the presence of a functional gene, and an exclamation mark (! ) indicates that the sequence is present but was evaluated to be non-functional. A cross (x) means no orthologous sequence was found. In addition, the position on chromosome or scaffold level of the self-incompatibility candidate genes in the genome is given. For the *Lolium perenne* L. genotype S23 Z and *Lolium multiflorum* Lam., both haplotypes of the diploid assemblies are given. *Triticum aestivum* L. represents an allohexaploid species leading to a triplication of the *S*- and *Z*-locus. Besides, on chromosome 2B (chr2B), a non-functional copy of the *ZDUF247-II* was present three times. In *Oryza longistaminata* A. Chev. & Roehr, the gene copies of functional *S* self-incompatibility candidate genes are present twice on two different scaffolds.

In the Poeae tribe, all SI candidate genes were present and assessed to be functional (fig. 6). Within the Poaceae family, orthologous genes of *sS* and *sZ* were mainly predicted to be functional, unlike most of the *SI-DUF247s* (fig. 6). Furthermore, all Poaceae species and genotypes investigated displaying a self-incompatible phenotype always harbored six functional SI candidate genes (fig. 6).

### Phylogenetic Analysis of Genes Located Within the *S*- and the *Z*-locus

**Fig. 7.**
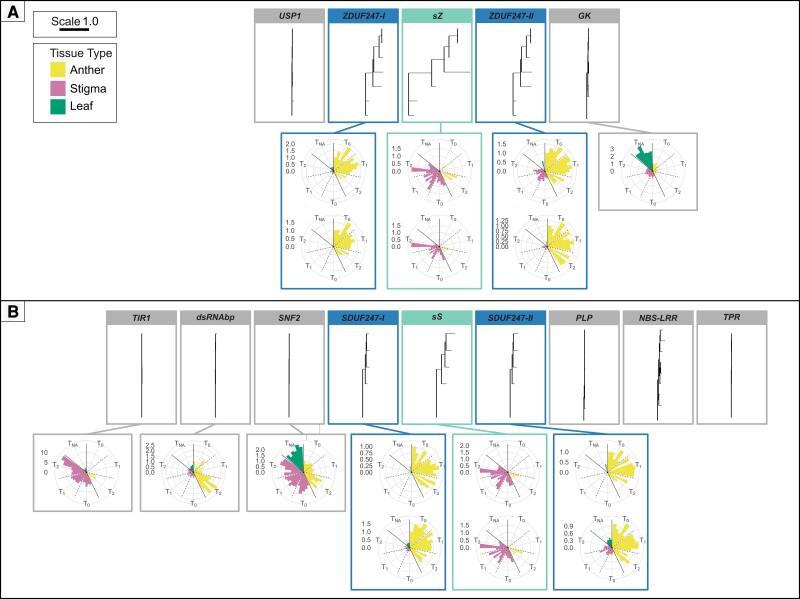
Phylogenetic trees and relative expression ratios of the genes at the *Z*-locus (A) and the *S*-locus (B). The phylogenetic trees and relative expression ratios are ordered according to the physical gene order as seen in *Lolium perenne* L. genotype Kyuss, and gene expression data is from the genotype S23 Z. The flanking genes are boxed in gray, whereas the self-incompatibility candidate genes are boxed in teal and blue. The scale bar in the top left corner represents one amino acid change per site, and the legend below shows the color code for the three tissue types used in the gene expression analysis. For the *LpsS*, *LpsZ*, and the *SI-DUF247s*, the relative expression ratio measurement was explicitly performed for each allele present in the genotype. The two different alleles are displayed stacked on top of each other. For the remaining *S*- and *Z*-locus genes, the expression pattern was investigated with primers amplifying both alleles, and therefore only one polar plot is presented per gene. Only data points were included where the *C_t_* difference was below 0.5, and the percent deviation was below 3% between the two technical replicates. Therefore, the standard error is not displayed in the figure.

The allelic richness of the genes within the *S*- and the *Z*-locus in *Lolium* spp. was evaluated using the coding sequences from the perennial ryegrass genotypes Kyuss, F1-30, P226/135/16, S23 Z, and the Italian ryegrass genotype Rabiosa. A phylogenetic tree was constructed for each gene using the alleles present (fig. 7). The SI candidate genes, as well as *NBS-LRR* and *LRR8*, exhibited a high allelic richness. The remaining genes at the *S*- and the *Z*-locus were highly conserved within the genus *Lolium*. To further investigate the sequence diversity of the SI candidate genes, a pairwise comparison based on a T-Coffee alignment of the AA sequence was performed and displayed in a heat map (supplementary fig. S1, Supplementary Material online). The sS alleles showed a mean protein sequence identity of 52.9% with a standard deviation (*σ*) of 6.8. The mean protein sequence identity of the sZ alleles was significantly lower, being 32.5% (*σ* = 9.5). The mean protein sequence identity between the sS and sZ alleles was 28.1% (*σ* = 4.3). The SI-DUF247s showed a higher level of protein sequence conservation within the different alleles, with the mean protein sequence identity being 79.5% (*σ* = 1.9) for SDUF247-I, 75.2% (*σ* = 3.2) for SDUF247-II, 60% (*σ* = 6.7) for ZDUF247-I, and 53% (*σ* = 6.9) for ZDUF247-II. A comparison between all homologs and genotypes of the SI-DUF247s revealed mean protein sequence identities ranging from 40.9% to 45.2%.

### Expression Analysis of Genes Located Within the *S*- and the *Z*-locus

To identify the female and the male components involved in the SI reaction, the expression pattern of the genes within the *S*- and the *Z*-locus in perennial ryegrass were analyzed using RT-qPCR. The expression pattern was investigated for the six SI candidate genes, one flanking gene at the *Z*-locus (*LpGK*) and three at *S*-locus (*LpTIR1*, *LpdsRNAbp*, and *LpSNF2*). Samples from the perennial ryegrass genotype S23 Z were taken from anther and stigma tissue at three development stages: 1 week before flowering (time point 0), 2–3 days before flowering (time point 1), and the day of flowering (time point 2). In addition, the leaf tissue of S23 Z was sampled with no specific time point (time point NA).

The high allelic diversity of *LpsS*, *LpsZ*, and the *LpSI-DUF247s* made it necessary to analyze both alleles for these genes individually. The expression data were visualized in a polar chart (fig. 7) and a scatter plot (supplementary fig. S2, Supplementary Material online) as the relative gene expression ratio calculated according to Pfaffl (2001). Moreover, the Δ*C_t_* values (*C_t_* value of the gene of interest minus the geometrical mean *C_t_* value of the reference genes) were calculated to allow the comparison of the expression levels of different genes within the same sample and are displayed in a heat map (supplementary fig. S3, Supplementary Material online).

Polar plots of the relative gene expression show that the *LpSI-DUF247s* genes displayed a tendency of anther-specific expression with decreasing expression toward the day of flowering. In leaf and stigma tissue, little or no *LpSDUF247* expression was measured (fig. 7 and supplementary figs. S2 and S3, Supplementary Material online). In contrast, the *LpsS* and *LpsZ* displayed a stigma-specific expression pattern. However, high expression was also measured in anther tissue for three biological replicates (A, C, and F) at time point 2 and the biological replicate E at time point 1 (supplementary figs. S2 and S3, Supplementary Material online). Furthermore, *LpsZ* and *LpsS* in stigma tissue displayed the highest expression levels relative to the reference genes (supplementary fig. S3, Supplementary Material online). *LpGK* and *LpSNF2* did not display a tissue-specific pattern and were expressed in all tissue types and development stages, with *LpGK* being overexpressed in leaves. The *LpdsRNAbp* showed an anther-specific expression with an apparent upregulation on the day of flowering. *LpTIR1* displayed stigma-specific expression according to the RT-qPCR experiment. *LpTIR1* showed up to a 14 times higher expression in the stigma on the day of flowering compared with the control sample (Anther T_1_ biological replicate A).

## Discussion

After almost 70 years of research on the two-locus gametophytic SI system of grasses, we established the gene content, order, and composition at the *S*- and the *Z*-locus. This is a major advancement since Manzanares et al. (2016) reported the identification of the putative male component at *S* (*LpSDUF247*). In that work, however, at least one component remained elusive in the absence of a contiguous genome sequence at the *S*-locus region. Similarly, at the *Z*-locus, only one *LpDUF247* gene was mentioned together with *LpTC116908* as prime candidates for SI determinants (Shinozuka et al. 2010). Our study further clarified the role of the genes identified previously and allowed the identification of two additional putative SI determinants, including the female components, at each locus.

For each locus, two male and one female determinant are suggested to govern the SI system in grasses. All four putative male determinants have a similar gene structure and encode for proteins belonging to the same family (DUF247). The putative female determinants at *S* (*sS*) and *Z* (*sZ*) are also of similar gene structure and are predicted to code for secreted proteins with no known family membership. Typical characteristics of SI determinants could be observed for the putative SI genes, including genetic and physical linkage, high allelic richness, high sequence diversity, and an anther- or stigma-specific expression pattern. Furthermore, the absence of a functional copy of at least one of the six putative SI determinants is accompanied by a self-compatible phenotype within the Poaceae species.

According to the hypothesis of Lundqvist (1962), the two-locus SI system in grasses originated from a duplication of a one-locus SI system. Following this hypothesis, the male and female determinants at *S* and *Z* represent gene duplicates with a similar gene sequence and structure (Yang et al. 2008). The *sZ*, *ZDUF247-I*, and *ZDUF247-II* at the *Z*-locus are the only three genes for which genes of similar sequence and structure were found at the *S*-locus (*sS*, *SDUF247-I*, and *SDUF247-II*) within self-incompatible grass species. The same protein family membership (DUF247), the same in silico motif predictions, similar protein size, and their conserved intronless gene structures are clear indicators of a shared origin for the *SI-DUF247s* genes within grasses. The presence of two *SI-DUF247s* genes at each locus also suggests that a duplication event within a SI locus occurred prior to the duplication of the whole locus. For the *sS* and *sZ*, the data also indicates a duplicative origin as both are of similar size, have the same gene structure (one intron), and have the same in silico protein motif prediction within self-incompatible grass species. Furthermore, the presence of one additional coding sequence similar to *SDUF247-II* outside of the *S*- and *Z*-locus indicates even a further duplication event. This additional *SDUF247-II* sequence is located on chromosome 6 in the reference-grade genome assembly of the perennial ryegrass genotype Kyuss without an annotation (position: 11313479...11315143).

The putative SI determinants at each locus were genetically and physically linked, indicating that they are inherited as a unit. The inheritance of SI determinants as a unit is necessary, as recombination events between SI determinants may lead to a breakdown of the SI system due to the generation of new haplotypes consisting of SI determinants expressing different SI specificities (Takayama and Isogai 2005). However, at both loci in self-incompatible grass species, the gene order and orientation vary, indicating preceding recombination events within the *S*- and *Z*-locus that, interestingly, did not lead to a breakdown of the SI system. Whereas unlikely due to the high quality of the genome assemblies used in this study, assembly errors at *S* and *Z* would represent an alternative explanation for the observed gene order and orientation changes.

Besides the unifying scheme that the SI determinants must be inherited as one segregating unit, the physiological reaction and SI genetics dictate that male determinants must be expressed in the pollen. In contrast, the female determinants must be expressed in the stigma (Takayama and Isogai 2005). The putative SI determinants, already being in line with the duplicative origin of the SI system in grasses and showing a close linkage, all displayed either an anther- or stigma-specific expression pattern. The *LpSI-DUF247s* having an anther-specific expression, and the *LpsS* and *LpsZ* a stigma-specific expression, allowing the conclusion that the *SI-DUF247s* represent the putative male SI determinants, whereas the *sS* and *sZ* represent the putative female determinant of the grasses SI system.

The *LpdsRNAbp* also showed anther-specific expression, whereas the *LpTIR1* showed a stigma-specific expression. Nonetheless, their direct involvement in the self-recognition process of the SI system in grasses can be excluded (*LpTIR1*) or is highly unlikely (*LpdsRNAbp*): The design of the mapping population used by Manzanares et al. (2016) for the fine-mapping of the *S*-locus dictated the presence of five alleles, and only three alleles were found for *LpTIR1*. Our analysis aligns with these findings as *TIR1* in the *Lolium* spp. analyzed showed high sequence conservation, uncharacteristic for an SI determinant. The same holds for the *dsRNAbp*, which, like *LpTIR1*, did not show a high allelic richness and sequence diversity within *Lolium* spp.. In addition, the argument can be brought forward that, for *dsRNAbp* and *TIR1*, no gene with a similar gene structure or sequence is present at both loci, contradicting the duplicative origin hypothesis of the two-locus SI system in grasses.

Besides the putative SI determinants being the only genes at the *S*- and the *Z*-locus suggesting a duplicative origin and at the same time showing an anther- or stigma-specific expression, they also showed the typical evolutionary characteristics of an SI determinant, that is, a high protein sequence diversity and a high allelic richness (Charlesworth et al. 2005). The observed protein sequence diversity of the putative SI determinants in *Lolium* spp. was comparable to the ones observed within the *S*-RNase type SI system (Ioerger et al. 1990; Ushijima et al. 1998; Williams et al. 2014; Dzidzienyo et al. 2016), the Papaveraceae type SI system (Paape et al. 2011), and the Brassicaceae type SI system (Jany et al. 2019). The sequence identities were matched best with the *S*-RNase type SI system with a highly diverse female determinant (*S*-RNase) (Ioerger et al. 1990; Ushijima et al. 1998; Dzidzienyo et al. 2016) and more conserved male determinants (*SLF*) (Williams et al. 2014). This is similar to the putative SI determinants in grasses, where the putative male determinants (*SI-DUF247*s) were more conserved than the female determinants (*sS* and *sZ*).

In addition to the high sequence diversity, the expected high allelic richness was also matched. Each allelic sequence of the putative SI determinants represents a unique allele with one exception at the *S*-locus and one exception at the *Z*-locus. The sequence diversity and allelic richness analysis were limited to the sequence data of four perennial and one Italian ryegrass genotype. A more representative picture can be seen when the data presented here is combined with additional sequence data available. Especially for the *LpSDUF247-I*, a total of 24 allele sequences could be identified, which showed a mean protein sequence identity of 78.5% (*σ* = 3.7) when our data was combined with the allelic sequences identified by Manzanares et al. (2016) and Veeckman et al. (2019).

Two more genes co-segregating with the *S*- or *Z*-locus would fulfill the requirement of high allelic richness and high sequence diversity: the *NBS-LRR* (*NBS-LRR-1*, *NBS-LRR-2*, and *NBS-LRR-3*) and the *LRR8*. Nonetheless, a possible SI determinant role is excluded for both: The involvement of the *NBS-LRR* in SI as an SI determinant was excluded in Manzanares et al. (2016) because the genes did not show a tissue-specific expression. Furthermore, the *NBS-LRR* was present as gene duplication or triplication within multiple *S*-loci, and the sequences were pooled for the sequence diversity and the allelic richness analysis, biasing the results. The *LRR8* is excluded as a possible SI determinant at the *Z*-locus because a marker with one recombination (BAC_BEG) lies within the gene’s coding sequence. Furthermore, for both the *NBS-LRR* and the *LRR8*, a gene with a similar gene structure and sequence is not present in both loci, contradicting the hypothesis of a duplicative origin of the two-locus SI system for grasses. Possible involvement in disease resistance was predicted for both genes, representing an alternative reason for the high allelic richness and the high sequence diversity observed (Mondragón-Palomino et al. 2002; McHale et al. 2006; Ng and Xavier 2011).

As additional evidence that the here reported putative SI determinants indeed govern the SI system in grasses, a distinctive genotypic pattern within the *S*- and *Z*-locus of self-incompatible and self-compatible genotypes can be seen. All self-incompatible grass genotypes have a functional copy of the *sS*, *SDUF247-I*, *SDUF247-II* at *S* and *sZ*, *ZDUF247-I*, and *ZDUF247-II* at *Z*. A genotype missing a functional copy in any of the putative SI determinants showed a self-compatible phenotype. The two *S. cereale* genotypes are worth mentioning as the genotype Weining displayed a predominantly outcrossing phenotype with a low selfing rate (indicating the presence of a leaky self-incompatibility system), and the inbred line Lo7 displayed a self-compatible phenotype. They differ genotypically as the inbred line Lo7 did not harbor a functional copy of the *ZDUF247-I*, representing an explanation for the breakdown of the SI system. In contrast, it cannot be concluded from a functional set of all six putative SI determinants that the plants show a self-incompatible phenotype. The absence of a functional gene copy of an SI determinant, disrupting the initial self-recognition process, is not the only source of self-compatibility (Do Canto et al. 2016). Similarly, a recombination event between the SI determinants, silencing of the SI determinants, or a mutation interfering with the downstream cascade of SI unlinked to the *S*- and the *Z*-locus represent other sources of self-compatibility (Do Canto et al. 2016; Cropano et al. 2021).

In our analysis, the perennial ryegrass genotype P226/135/16 represented the only case where six functional SI determinants were present but no self-incompatible phenotype was reported. The source of self-compatibility for that genotype remains unknown. But the loss of close linkage of the putative SI determinants at the *Z*-locus indicates a recombination event and a possible self-compatibility source (Takayama and Isogai 2005).

Based on the physiological observations of the pollen tube growth and its halt in self-incompatible grass species, Heslop-Harrison and Heslop-Harrison (1982) suggested that the male determinant must be anchored in the membrane of the pollen and that the female determinant is a secreted and diffusible protein. A similar mechanism of the SI system was also suggested by Wehling et al. (1994). The putative male determinants (SI-DUF247s) being predicted to be membrane-bound proteins and the putative female determinants (sS and sZ) predicted to be secreted into the extracellular space would agree with the suggested physiological mechanisms. The identification of the putative SI determinants on the AA sequence level also allows us to further speculate about the mode of action of the SI system in grasses. It is plausible that the two SDUF247s and the two ZDUF247s each form a heterodimer, representing a receptor toward its ligand sS and sZ, respectively. The interaction of an SI-DUF247 heterodimer with its female determinant of the same SI specificity would trigger an unknown signal. If this unknown signal accumulates from both loci *S* and *Z*, a downstream reaction is triggered, leading to the halt of the pollen tube growth. Furthermore, it is also plausible that all four SI-DUF247s would form a heterotetramer. The interaction of the SI-DUF247 heterotetramer with both the sS and sZ of the same SI specificity would trigger a downstream reaction, leading to the halt of the pollen tube growth. In order to elucidate the mode of action of the SI system in grasses and to test the proposed hypotheses, it will be crucial to functionally characterize the putative SI determinants in vivo using, for example, bimolecular fluorescence complementation (BiFC) or co-immunoprecipitation (Co-IP).

Whereas our analysis was mainly focused on *Lolium* spp., it is commonly believed that the outbreeding nature of grass species can be attributed to the same SI system (Li et al. 1997; Baumann et al. 2000). This belief is further supported by the high synteny observed of the *S*- and *Z*-locus and especially the presence of functional copies of the putative SI determinants in self-incompatible species belonging to the Poeae tribe (*L. perenne*, *L. multiflorum*, and *D. glomerata*), Triticeae tribe (*S. cereale*), and Oryzeae tribe (*O. longistaminata*). Furthermore, for another member of the Triticaceae tribe (*H. bulbosum*), the gene *HPS10* was presented as a possible candidate for the female determinant at the *S*-locus (Kakeda et al. 2008; Kakeda 2009). The *HPS10* is orthologous to the presented putative female determinant at the *S*-locus (*sS*). Our findings further support those of Lian et al. (2021), who reported two male candidates at the *S*-locus, *OlSS1* and *OlSS2*, orthologous toward the *SDUF247-I* and *SDUF247-II*, and one female candidate, the *OlSP*, orthologous to the *sS*.

In conclusion, our study provides multiple lines of evidence that the *SI-DUF247* are the male SI determinants in grasses at both loci (*S* and *Z*), whereas *sS* is the female determinant at *S*, and *sZ* is the female determinant at *Z*. The identification of the SI determinants enables the prediction of pollen compatibility and pollination efficiency as well as the targeted induction and exploitation of loss-of-function mutations at *S* or *Z*, leading to self-compatibility, both a quantum leap in the breeding of allogamous grass species. More broadly, our study offers new insights into the origin and evolution of the unique gametophytic SI system in one of the largest and economically most important plant family.

## Materials and Methods

### Fine-mapping of the *Z*-locus in Perennial Ryegrass

The two perennial ryegrass populations used for fine-mapping (VrnA-XL and DTZ) were designed to segregate for the *Z*-locus as described by Manzanares et al. (2016). VrnA-XL had its origin in the VrnA population (Jensen et al. 2005), initially derived from a cross between a genotype of the Italian cv. ’Veyo2’ and an ecotype collected on the Danish island Falster. The F_1_ genotype F1-30 (SI composition S_12_Z_22_) was clonally propagated at a large scale and pollinated with pollen from a second F_1_ genotype (F1-39, S_12_Z_12_). The resulting offspring, harvested on F1-30, were supposed to be heterozygous at the *Z*-locus. Homozygosity at the *Z*-locus either indicated rarely occurring self-pollination or a recombination event between the marker under investigation and the *Z*-locus. Similarly, DTZ originated from the perennial ryegrass ILGI mapping family (Jones et al. 2002) and was developed by crossing the ILGI siblings P150/112/129 (*S*_12_*Z*_13_) and P150/112/132 (*S*_12_*Z*_12_) but in the opposite direction as reported in Manzanares et al. (2016), that is, P150/112/129 as male and P150/112/132 as the female parent.

Single seeds of both VrnA-XL and DTZ were grown in soil-filled plastic trays (8 × 12 pots), covered by a thin layer of sand. Around 4 weeks after germination, young leaf samples (approximately 15 cm long) were collected in 96-well collection plates and used for high-throughput DNA extraction as described in Manzanares et al. (2016).

For fine-mapping and BAC library screening, publicly available DNA markers from Hackauf and Wehling (2005) and Shinozuka et al. (2010) were used. Additional markers were developed by alignment of P205C9H17P and additional BAC clone sequences kindly provided by Prof. Iain Armstead, later published by Harper et al. (2019), with the rice genome sequence (RAP Build 3 *of O. sativa japonica*, NCBI) using BLASTN analysis. Primers were designed in regions being conserved between rice and perennial ryegrass using the Primer3 software (Untergasser et al. 2012). Markers were designed to amplify PCR products of 80–150 bp, suitable for high-resolution melting (HRM) analysis of unknown DNA sequence polymorphisms as described by Studer et al. (2009). Genotyping of VrnA-XL and DTZ was done at high throughput using HRM analysis as described by Manzanares et al. (2016).

### Construction of the Poaceae Synteny Maps and Assessment of Functionality of the Candidate Genes

The gene annotations from the perennial ryegrass genotype Kyuss (Frei et al. 2021), the perennial ryegrass genotype P226/135/16 (Begheyn et al. 2018), and the genome assembly of the Italian ryegrass genotype Rabiosa (Copetti et al. 2021) were used to obtain the sequences of the genes within the *S*-locus and the *Z*-locus. For this purpose, the flanking markers of the *S*-locus (05_02790 and 05_02889, Manzanares et al. 2016) and the *Z*-locus (CADELP, 37600, BAC_BEG, 171R) were used to identify the *S*- and the *Z*-locus, respectively. Gene annotations were considered if an orthologous sequence was present within the *S*-locus and *Z*-locus of Kyuss, P226/135/16, and Rabiosa; otherwise, they were removed from further analysis. If no annotation was present for an identified coding sequence, the gene structure was added using the Augustus (Organism: *Oryza brachyantha* L.) gene prediction tool or manually through a BLAST-based approach (Stanke and Morgenstern 2005). Furthermore, the intron–exon structure leading to the coding sequence was further streamlined for all the genes in the three genome assemblies using the ORF finder from NCBI combined with a BLAST-based manual approach. Therefore, the coding sequence based on the annotation file does not always perfectly align with the coding sequence used in this study. For example, within the three high-quality genome assemblies displayed in table 1, 16 *SI-DUF247* gene sequences were identified. Of these 16 identified gene sequences, only four were annotated as an intronless gene, whereas the other sequences were either not annotated or annotated with minor or major deviations toward the intronless gene structure. All 16 gene sequences identified were then streamlined into an intronless gene structure of similar size.

Using the CLC Genomics Workbench software 11.0 (CLC bio, Aarhus, Denmark), the identified *S*- and *Z*-locus genes and flanking marker amplicon sequences were used as a query for BLAST analysis against a database containing 13 Poaceae genome assemblies (table 2). BLAST hits were mapped if the BLAST E-value was below 1 × 10^−80^ for all the genes except *sS* and *sZ*. For *sS* and *sZ*, the BLAST E-value was 1 × 10^−10^. Furthermore, when a new orthologous sequence of *sS* or *sZ* was identified, it was added to the BLAST query. For the amplicon sequences of the flanking markers, the BLAST E-value was 1 × 10^−10^. The BLAST-based annotation files of the *S*- and *Z*-locus were then translated into a CMAP file format as Veltri et al. (2016) described. The CMAP files were used to generate synteny maps using the advanced mode of the SimpleSynteny tool (Veltri et al. 2016). The graphical representation (e.g., fill colors of the structures) of the synteny maps was adapted using the Affinity Designer software (Serif (Europe) Ltd, West Bridgford, UK). In order to assess the functionality of the SI candidate genes, besides standard analysis regarding the size of a protein and the number of introns, InterProScan was used to predict the protein motifs (Blum et al. 2021; https://www.ebi.ac.uk/interpro/search/sequence).

### Phylogenetic Tree Construction of *S*- and *Z*-locus Genes

The *S*- and *Z*-locus gene sequences from the perennial ryegrass genotypes Kyuss, F1-30 (haplotype P205), P226/135/16, and S23 Z, as well as from the Italian ryegrass genotype Rabiosa, were extracted. The intron–exon structure of all *S*- and *Z*-locus genes extracted were further streamlined, leading to a comparable gene structure using the ORF finder from NCBI combined with a BLAST-based manual approach. The *LmTPR-2* of the Rabiosa scf818 (*Lmu01_818G0000280*) was excluded from analysis, as it represented a distinctive duplication to the *TPR-1* and was present only once in the six *S*-locus regions analyzed. The *LpGK-2* from P226/35/16 was excluded as it seemed to display a truncated duplication of the *GK-1*, and no streamlined coding sequence could be found. The coding sequence for 44 *S*-locus and 75 *Z*-locus gene sequences were extracted. The duplications of *NBS-LRR*, *GK*, and *PLP* were pooled. TranslatorX was used for AA-directed multiple sequence alignment for each group of orthologous genes (Abascal et al. 2010) by using the MAFFT algorithm v7.147b (Katoh et al. 2002). An additional alignment curation step was performed using Gblocks v0.91b with the minimal block length of five AAs (Talavera and Castresana 2007). The alignment file was then transformed into the PHYLIP format using EasycodeML v1.2 (Gao et al. 2019). The phylogenetic trees were built using PhyML-3.1 (Guindon et al. 2010). The trees were visualized using the ggtree package in R statistical environment, version 4.1.1 (Yu 2020).

### Pairwise Comparison of Protein Sequences of the Putative SI Determinants

A multiple protein sequence alignment of the *Lolium* male SI candidate proteins (SI-DUF247s) and the *Lolium* female SI candidate proteins (sS and sZ) was performed using the T-Coffee multiple sequence alignment package provided by EMBL-EBI (Madeira et al. 2019). The calculated percentage identity matrix was converted into a heat map for graphical representation.

### Expression Pattern Analysis of *S*- and *Z*-locus Genes Using RT-qPCR

#### Plant Material and Growth Conditions

The self-incompatible and highly heterozygous perennial ryegrass genotype S23 Z (Valentine and Charles 1975) was vernalized over the winter outdoors in Eschikon, Switzerland. A total of six clones were transferred into a climate chamber in the spring once the first signs of flowering (emerging of flower heads) were visible. The plants were grown under long-day conditions (16 h light; 8 h dark) with temperatures ranging from 20 °C during the night and 24 °C during the day.

#### Tissue Sampling

Anther and stigma tissue were collected during flowering at three different time points (T_0_: 1 week before flowering, T_1_: 2–3 days before flowering, T_2_: on the day of flowering). Leaf tissue was collected as a control and did not have a specific time point (T_NA_). The sampled tissue was transferred in a 1.5 ml Eppendorf tube, immediately frozen in liquid nitrogen, and stored in a −80 °C freezer. Anther tissue was collected instead of pollen tissue as a sufficient amount of pollen for RNA extraction prior to the day of flowering (T_2_) is not possible with perennial ryegrass.

#### RNA Extraction and cDNA Synthesis

Plastic grinding pestles were used to homogenize 45–80 mg of plant tissue in a 1.5 ml Eppendorf tube. The ground tissue was used for RNA extraction using the Qiagen RNeasy Mini Kit, following the “Purification of Total RNA from Plant Cells and Tissues and Filamentous Fungi” protocol (Qiagen, Hilden, Germany). Furthermore, an additional on-column DNase Digestion with the RNAse-Free DNase Set was performed according to the manufacturer’s protocol (Qiagen, Hilden, Germany). The integrity of the total RNA extracted was confirmed with the TapeStation 2200 using RNA screen tape (Agilent Technologies, Santa Clara, CA, USA). RNA samples with an RNA integrity number (RIN) value below 4.5 were discarded (Schroeder et al. 2006). The RNA quantity was determined using the Qubit BR RNA assay (Thermo Fischer Scientific, Waltham, MA, USA). The weight, the concentration in ng/μl, and the individual RIN values can be seen in supplementary table S3, Supplementary Material online. Double-stranded cDNA was synthesized from 0.3 to 1 μg of RNA using the RevertAid First Strand cDNA Synthesis Kit (Thermo Fischer Scientific, Waltham, MA, USA), following the manufacturer’s protocol with 0.5 μl Oligo (dT)18 primer and 0.5 μl Random Hexamer primers. For each RNA sample that was reverse transcribed (RT sample), a no-reverse transcriptase control (NoRT sample) was included to detect a possible genomic DNA contamination of the RNA samples.

#### Primer Design

Primer pairs were designed for multiple genes of interest (GOI) co-segregating with the *S*- and the *Z*-locus of perennial ryegrass (supplementary table S2, Supplementary Material online). Furthermore, primer pairs were designed for the reference genes *EF1-α*, *elF4A-2*, *CPB20*, and *elf4A-1* as they showed a conserved expression level between pollen and stigma samples (Manzanares et al. 2016). The unresolved diploid genome assembly of the perennial ryegrass genotype S23 Z was used to obtain the sequences of the GOI and the reference genes. The software Primer3 (Untergasser et al. 2012) was used to design primers leading to a product size of 75–160 bp, a primer melting temperature of 60 °C, and a primer size of 18–23 bp. The *SDUF247-I*, *SDUF247-II*, *LpsS*, *ZDUF247-I*, *ZDUF247-II*, and *LpsZ* displayed a high sequence diversity between the two alleles, making it necessary to design allele-specific primers. The sequences of the primers for the amplification of the GOI and the reference genes are displayed in supplementary table S2, Supplementary Material online.

#### RT-qPCR Data Acquisition

The RT-qPCR was performed on the high-throughput BioMark HD system using a 192.24 Dynamic Array (Fluidigm, South San Francisco, CA, USA). All of the samples were pre-amplified for 17 cycles before the initial run, following the manufacturer’s protocol, and the final product was diluted fivefold. The samples and four negative control samples (ddH_2_O) were loaded in duplicates according to Fluidigm’s EvaGreen DNA-binding dye protocol onto the BioMark HD system. The following qPCR conditions were used: 95 °C for 30 s, 40 cycles of 95 °C for 5 s, and 60 °C for 20 s, plus a melting curve analysis. The data were processed using the software Fluidigm Real-Time PCR analysis 4.0 (Fluidigm, South San Francisco, CA, USA). The quality threshold was set to the default value of 0.65. Furthermore, a linear baseline correction was performed, and the setting automatic detectors were used as the *C_t_* threshold method.

#### RT-qPCR Data Analysis

The *C_t_* values were exported using the Fluidigm Real-Time PCR analysis 4.0 software (Fluidigm, South San Francisco, CA, USA). All 16 GOI and the four reference genes consistently showed a single amplicon peak. Measured *C_t_* values over 21 were set to 999. This specific threshold was chosen as we observed a high standard deviation between the technical replicates with values above 21. The standard deviation between the two technical replicates for each sample was calculated, and data points with more than a 0.5 *C_t_* difference were excluded from downstream analysis. Besides, only data were used where the percent deviation between the two technical replicates was below 3%. Furthermore, the four ddH_2_O controls were assessed for each primer pair to exclude possible contamination in the chemicals. The gDNA contamination was assessed by comparing the RT samples’ *C_t_* value with the noRT samples’ *C_t_* value for each of the four reference genes. The gDNA contamination was considered negligible when the difference in *C_t_* value between the RT sample and the noRT sample was above ten cycles. The primer efficiencies were calculated using LinReg PCR 7.5 (Ramakers et al. 2003) and are shown in supplementary table S2, Supplementary Material online. The stability of the reference genes was assessed using GeNorm (Huang et al. 2014). The expression stability value for *EF1-alpha* was 0.292, for *CPB20* was 0.488, for *eIF4A-2* was 0.292, and for *eIF4A-1* was 0.385, meaning all four reference genes qualify to be used as they all have an expression stability value below 1.5 which represent the geNorm cut off (Vandesompele et al. 2002).

To determine the relative gene expression ratio of the GOI, a relative quantification method, as described by Pfaffl (2001), was used. The Δ*C_t_* value was calculated by subtracting the *C_t_* value of the sample minus the *C_t_* value of the control. *LpTIR*, *LpdsRNAbp*, *SNF2*, *LpGK*, *LpSDUF247-I*, *LpSDUF247-II*, *LpZDUF247-I*, and *LpZDUF247-II*, the first biological replicate of the anther tissue at time point 1 was used as the control. For *LpsS* and *LpsZ*, the first biological replicate of the stigma tissue at time point 1 was used as the control. The Δ*C_t_* was set to the power of the respective PCR efficiency, leading to the relative quantity (RQ) value. The relative gene expression was calculated by dividing the RQ value of the GOI by the geometrical mean RQ value of the reference gene. The relative gene expression ratios of the *S*- and *Z*-locus genes were displayed in polar charts and a scatter plot using the R statistical environment, version 4.1.1.

In addition, the Δ*C_t_* values of *S*- and *Z*-locus genes were calculated to compare the expression levels of different genes in the same sample. The Δ*C_t_* values were calculated as the *C_t_* value of GOI minus the geometrical mean *C_t_* value of the four reference genes (*EF1-alpha*, *CPB20*, *eIF4A-2*, and *eIF4A-1*). The Δ*C_t_* values were then displayed in a heat map using the R statistical environment, version 4.1.1. No scaling was applied, and the data were clustered on the level of genes (rows).

## Supplementary Material

msac259_Supplementary_Data

## Data Availability

The nucleotide sequence of the BAC clone P205C9H17P is available under the GenBank accession number OP292309. The nine contigs spanning the *S*- and the *Z*-locus of the perennial ryegrass genotype S23 Z are available under the GenBank accession numbers OP292310, OP292311, OP292312, OP292313 OP292314, OP292315, OP292316, OP292317, and OP292318. The nucleotide sequence of the four scaffolds spanning the *S*- and the *Z*-locus of the Italian ryegrass genotype Rabiosa are available at http://doi.org/10.5281/zenodo.7289792. The corresponding annotation file is available at http://doi.org/10.5281/zenodo.7015164. The coding sequence of all genes identified within the *S*- and *Z*-locus within *Lolium* spp. are available at http://doi.org/10.5281/zenodo.7290695.
