# Supplementary material for: Fine-Mapping and Comparative Genomic Analysis Reveal the Gene Composition at the S and Z Self-incompatibility Loci in Grasses
Source: Mol Biol Evol. 2022 Dec 8;40(1):msac259. doi: 10.1093/molbev/msac259 (PMC9825253; doi:10.1093/molbev/msac259)
Supplement: msac259_Supplementary_Data [file msac259_supplementary_data.pdf]

Supplementary material

A

Species and genotype	Localization	Protein name	1.	2.	3.	4.	5.	6.	7.	8.	9.	10.	11.	12.	13.	14.	15.	16.	17.	18.	19.	20.	21.	22.	23.	24.	25.	26.
			SDUF247-I						SDUF247-II						ZDUF247-I							ZDUF247-II						
1. *L. perenne* Kyuss	chr1	SDUF247-I	100	80.72	78.81	81.85	78.81	77.36	43.02	42.77	41.01	43.53	41.01	42.26	45.69	43.46	42.88	43.46	42.91	44.38	42.52	41.38	41.84	41.15	41.84	38.96	41.17	40.73
2. *L. perenne* P226/135/16	chr1		80.72	100	77.23	81.36	77.23	79.85	42.75	42.5	41.31	44.23	41.31	42.94	46.97	43.19	43.57	43.19	42.64	44.29	42.64	40.54	41.57	41.35	41.57	38.12	39.15	40.66
3. *L. perenne* S23 Z	contig11029		78.81	77.23	100	78.02	100	82.61	43.5	43.43	42.6	43.9	42.6	44.49	43.82	42.38	42.97	42.38	43.4	43.71	42.54	41.78	42.25	41.65	42.25	40.04	41.33	42
4. *L. perenne* S23 Z	contig12948		81.85	81.36	78.02	100	78.02	79.1	44.1	44.23	42.47	45.4	42.47	44.08	46.77	44.72	44.15	44.72	43.6	44.68	43.8	41.49	42.72	40.96	42.72	38.89	41.28	41.23
5. *L. multiflorum*	scf1212		78.81	77.23	100	78.02	100	82.61	43.5	43.43	42.6	43.9	42.6	44.49	43.82	42.38	42.97	42.38	43.4	43.71	42.54	41.78	42.25	41.65	42.25	40.04	41.33	42
6. *L. multiflorum*	scf818		77.36	79.85	82.61	79.1	82.61	100	42.08	42.42	40.85	43.16	40.85	42.48	45.31	43.3	43.49	43.3	41.59	42.47	42.17	39.31	40.15	39.35	40.15	38.05	40.04	39.62
7. *L. perenne* Kyuss	chr1	SDUF247-II	43.02	42.75	43.5	44.1	43.5	42.08	100	76.18	82.18	75.85	82.18	72.45	46.84	43.41	42.83	43.41	45.42	45.61	46.86	38.54	42.69	42.47	42.69	41.15	40.96	41.36
8. *L. perenne* P226/135/16	chr1		42.77	42.5	43.43	44.23	43.43	42.42	76.18	100	74.59	76.23	74.59	72.5	47.64	44.51	44.89	44.51	46.71	46.71	46.98	40.04	43.68	42.12	43.68	41.49	41.11	41.31
9. *L. perenne* S23 Z	contig11029		41.01	41.31	42.6	42.47	42.6	40.85	82.18	74.59	100	74.91	100	71.58	47.04	43.13	42.55	43.13	46.11	45.72	46.97	37.5	41.92	40.73	41.92	42.03	42.03	41.67
10. *L. perenne* S23 Z	contig11774		43.53	44.23	43.9	45.4	43.9	43.16	75.85	76.23	74.91	100	74.91	72.97	47.52	44.81	42.86	44.81	45.67	46.26	47.14	40.08	43.77	42.38	43.77	42.41	42.02	41.76
11. *L. multiflorum*	scf1212		41.01	41.31	42.6	42.47	42.6	40.85	82.18	74.59	100	74.91	100	71.58	47.04	43.13	42.55	43.13	46.11	45.72	46.97	37.5	41.92	40.73	41.92	42.03	42.03	41.67
12. *L. multiflorum*	scf818		42.26	42.94	44.49	44.08	44.49	42.48	72.45	72.5	71.58	72.97	71.58	100	47.08	42.48	42.48	42.48	45.4	46.55	45.66	39.28	42.61	40.87	42.61	40.72	42.45	41.49
13. *L. perenne* Kyuss	chr2	ZDUF247-I	45.69	46.97	43.82	46.77	43.82	45.31	46.84	47.64	47.04	47.52	47.04	47.08	100	58.22	57.84	58.22	57.25	58.75	59.36	45.16	47.1	48.16	47.1	46.36	45.29	45.02
14. *L. perenne* F1-30	P205		43.46	43.19	42.38	44.72	42.38	43.3	43.41	44.51	43.13	44.81	43.13	42.48	58.22	100	73.62	100	55.7	56.24	58.61	42.3	43.29	43.73	43.29	43.71	42.42	43.9
15. *L. perenne* P226/135/16	chr2		42.88	43.57	42.97	44.15	42.97	43.49	42.83	44.89	42.55	42.86	42.55	42.48	57.84	73.62	100	73.62	54.02	54.38	57.68	43.41	43.1	43.54	43.1	41.09	41.65	44.09
16. *L. perenne* S23 Z	contig55097		43.46	43.19	42.38	44.72	42.38	43.3	43.41	44.51	43.13	44.81	43.13	42.48	58.22	100	73.62	100	55.7	56.24	58.61	42.3	43.29	43.73	43.29	43.71	42.42	43.9
17. *L. perenne* S23 Z	contig7728		42.91	42.64	43.4	43.6	43.4	41.59	45.42	46.71	46.11	45.67	46.11	45.4	57.25	55.7	54.02	55.7	100	79	57.47	42.29	44.57	44.25	44.57	44.4	43.13	43.07
18. *L. multiflorum*	scf1905		44.38	44.29	43.71	44.68	43.71	42.47	45.61	46.71	45.72	46.26	45.72	46.55	58.75	56.24	54.38	56.24	79	100	58.76	42.51	45.44	44.36	45.44	43.86	43.71	44.61
19. *L. multiflorum*	scf3448		42.52	42.64	42.54	43.8	42.54	42.17	46.86	46.98	46.97	47.14	46.97	45.66	59.36	58.61	57.68	58.61	57.47	58.76	100	42.29	42.53	44.32	42.53	42.69	43.5	43.07
20. *L. perenne* Kyuss	chr2	ZDUF247-II	41.38	40.54	41.78	41.49	41.78	39.31	38.54	40.04	37.5	40.08	37.5	39.28	45.16	42.3	43.41	42.3	42.29	42.51	42.29	100	49.43	50.47	49.43	50.28	50.47	51.39
21. *L. perenne* F1-30	P205		41.84	41.57	42.25	42.72	42.25	40.15	42.69	43.68	41.92	43.77	41.92	42.61	47.1	43.29	43.1	43.29	44.57	45.44	42.53	49.43	100	67.04	100	50.28	51.06	50.86
22. *L. perenne* P226/135/16	chr2		41.15	41.35	41.65	40.96	41.65	39.35	42.47	42.12	40.73	42.38	40.73	40.87	48.16	43.73	43.54	43.73	44.25	44.36	44.32	50.47	67.04	100	67.04	49.81	47.97	50.67
23. *L. perenne* S23 Z	contig55097		41.84	41.57	42.25	42.72	42.25	40.15	42.69	43.68	41.92	43.77	41.92	42.61	47.1	43.29	43.1	43.29	44.57	45.44	42.53	49.43	100	67.04	100	50.28	51.06	50.86
24. *L. perenne* S23 Z	contig4538		38.96	38.12	40.04	38.89	40.04	38.05	41.15	41.49	42.03	42.41	42.03	40.72	46.36	43.71	41.09	43.71	44.4	43.86	42.69	50.28	50.28	49.81	50.28	100	73.5	49.44
25. *L. multiflorum*	scf1905		41.17	39.15	41.33	41.28	41.33	40.04	40.96	41.11	42.03	42.02	42.03	42.45	45.29	42.42	41.65	42.42	43.13	43.71	43.5	50.47	51.06	47.97	51.06	73.5	100	48.76
26. *L. multiflorum*	scf3448		40.73	40.66	42	41.23	42	39.62	41.36	41.31	41.67	41.76	41.67	41.49	45.02	43.9	44.09	43.9	43.07	44.61	43.07	51.39	50.86	50.67	50.86	49.44	48.76	100

B

Species and genotype	Localization	Protein name	1.	2.	3.	4.	5.	6.	7.	8.	9.	10.	11.	12.	13.
			sS						sZ						
1. *L. perenne* Kyuss	chr1	sS	100	52.44	62.65	48.15	62.65	57.32	30.16	30.16	27.94	20	30.16	22.95	24.62
2. *L. perenne* P226/135/16	chr1		52.44	100	53.57	55	53.57	60.71	35.29	30.88	31.51	21.54	30.88	24.62	24.29
3. *L. perenne* S23 Z	contig11029		62.65	53.57	100	39.51	100	51.19	29.23	30.77	26.76	22.58	30.77	24.19	19.4
4. *L. perenne* S23 Z	contig12948		48.15	55	39.51	100	39.51	53.09	30.65	32.26	37.31	33.9	32.26	33.33	32.81
5. *L. multiflorum*	scf1212		62.65	53.57	100	39.51	100	51.19	29.23	30.77	26.76	22.58	30.77	24.19	19.4
6. *L. multiflorum*	scf818		57.32	60.71	51.19	53.09	51.19	100	27.69	29.23	32.86	25.81	29.23	24.19	26.87
7. *L. perenne* Kyuss	chr2	sZ	30.16	35.29	29.23	30.65	29.23	27.69	100	31.08	34.18	35.62	31.08	32.43	29.73
8. *L. perenne* F1-30	P205		30.16	30.88	30.77	32.26	30.77	29.23	31.08	100	53.26	24.39	100	24.39	28.05
9. *L. perenne* P226/135/16	chr2		27.94	31.51	26.76	37.31	26.76	32.86	34.18	53.26	100	28.41	53.26	21.84	34.52
10. *L. perenne* S23 Z	contig4358		20	21.54	22.58	33.9	22.58	25.81	35.62	24.39	28.41	100	24.39	53.75	30.26
11. *L. perenne* S23 Z	contig55097		30.16	30.88	30.77	32.26	30.77	29.23	31.08	100	53.26	24.39	100	24.39	28.05
12. *L. multiflorum*	scf1905		22.95	24.62	24.19	33.33	24.19	24.19	32.43	24.39	21.84	53.75	24.39	100	26.32
13. *L. multiflorum*	scf3448		24.62	24.29	19.4	32.81	19.4	26.87	29.73	28.05	34.52	30.26	28.05	26.32	100

C

100
90
80
70
60
50
40
30
20

Supplementary Figure 1: Pairwise comparison of the protein sequences of the male and female self-incompatibility candidates. (A) Pairwise comparison for the SI-DUF247s. (B) Pairwise comparison for the sS and sZ. (C) Color code indicating the protein sequence identity in percentage.

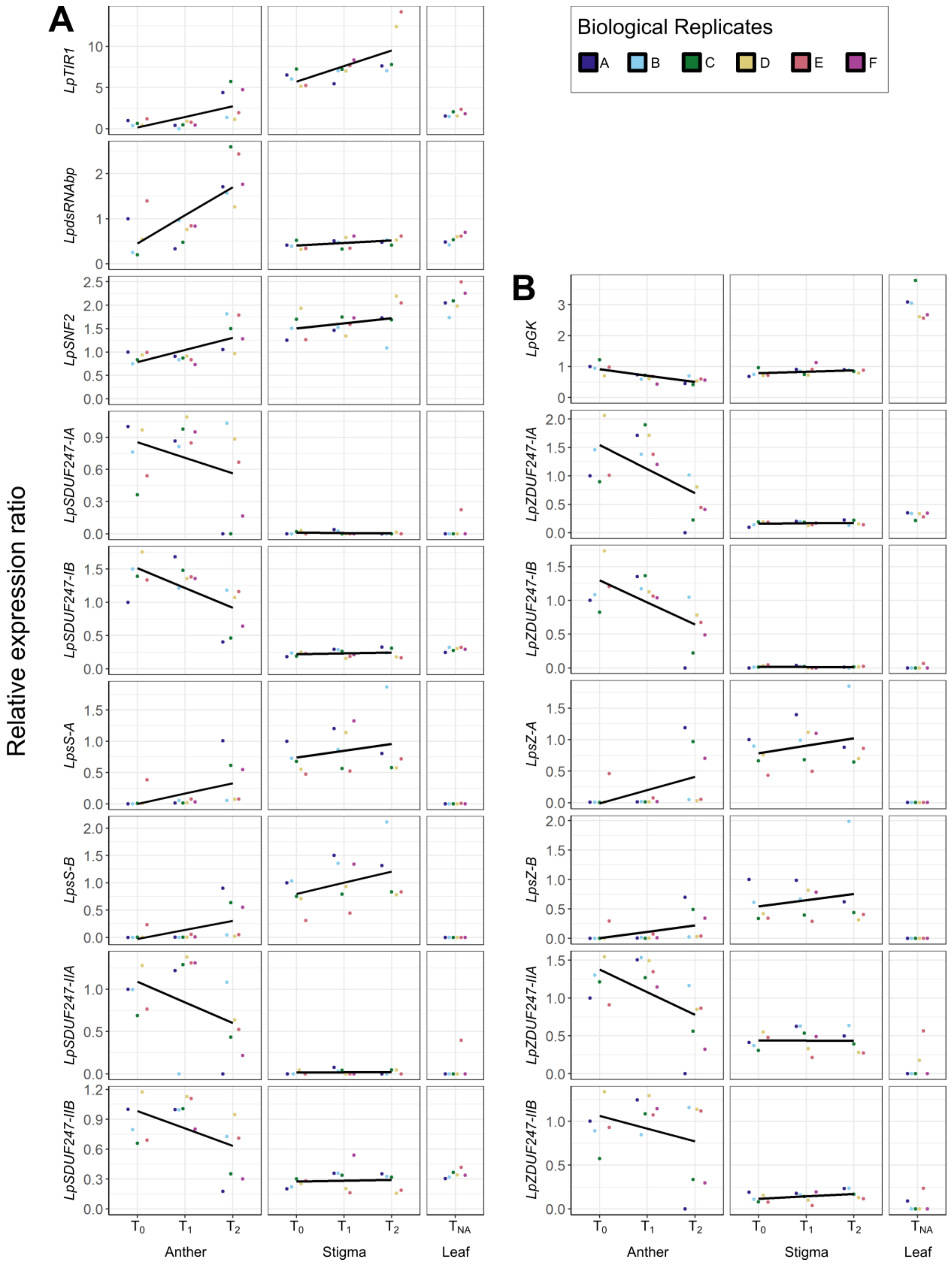

Supplementary Figure 2: Relative expression ratio of *S*- and *Z*-locus genes in *Lolium perenne* L. genotype S23 Z. The relative expression ratios were calculated according to Pfaffl (2001) and normalized to four reference genes (*EF1-alpha*, *CPB20*, *eIF4A-2*, *eIF4A-1*). For *LpsS* and *LpsZ*, the expression value in stigma tissue at time point 0 (T_0) in the first biological replicate was set as 1. For all the other genes displayed, the expression value in anthers at time point 0 (T_0) in the first biological replicate was set to 1. The color scheme in the top right corner is used to distinguish the biological replicates. The relative expression ratios can be seen on the Y-axis and the different sample tissues at different time points on the X-axis. Furthermore, for the *LpsS*, *LpsZ*, and the *SI-DUF247*s, the relative expression ratio measurement was explicitly performed for each allele present in the genotype. The two different alleles are indicated by A and B after the gene name. Only data points were included, where the C_t difference was below 0.5 and the percent deviation was below 3% between the two technical replicates. Therefore, the standard error is not displayed in the figure. (A) Relative expression ratios of *S*-locus genes. (B) Relative expression ratios of *Z*-locus genes.

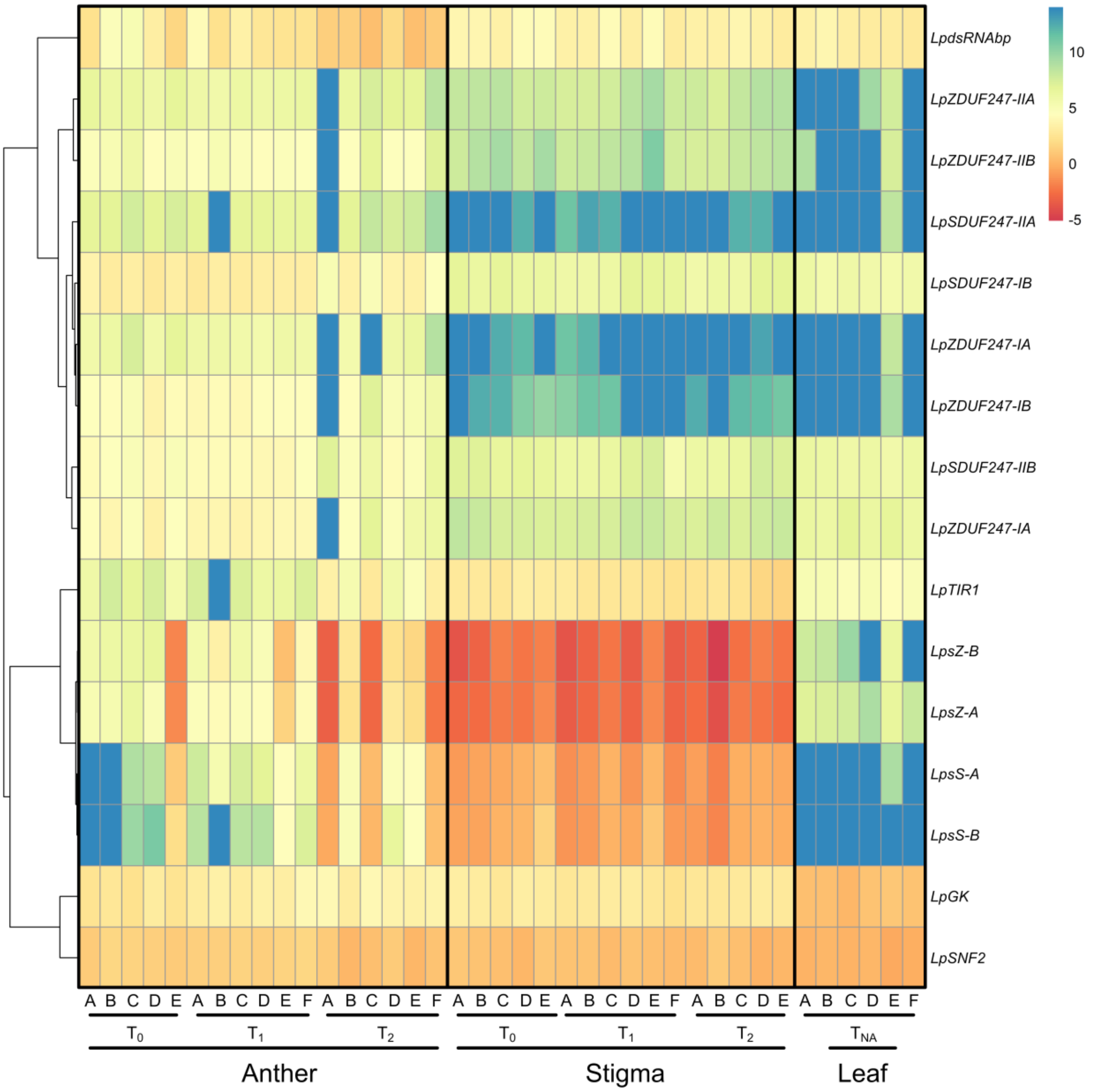

Supplementary Figure 3: Expression levels of *S*- and *Z*-locus genes relative to four reference genes in *Lolium perenne* L. genotype S23 Z. The ΔC_t values were calculated as the C_t value of the gene of interest minus the geometrical mean C_t value of the four reference genes (*EF1-alpha, CPB20, eIF4A-2, eIF4A-1)*. The rows indicate the different genes localized within the *S* and *Z* loci. For the *LpsS*, *LpsZ* and the *SI-DUF247*s, the ΔC_t was calculated for both alleles present in the genotype. The two different alleles are indicated by A and B after the gene name. The columns indicate the different sample types at different time points. The biological replicates are indicated with A-F. No scaling was applied, and the data were clustered on the level of genes (rows). A dendrogram is drawn on the left, illustrating the hierarchical relationship between the expression pattern of the genes. Blue corresponds to low expression, whereas red corresponds to high expression.

Supplementary Table 1: Number of the recombination events observed between molecular markers at the *Z*-locus region in the VrnA-XL and DTZ population.

Marker names	Forward primer sequence	Reverse primer sequence	VrnA-XL	DTZ
CADELP	TGCTCTTGAGGTTTGGCTAT	ATTGAGTCTTCAACAGCATTTTT	22	48
37600	AAGTGGGACAAAAGGGAACC	ATGAAGTACAAGCAAGAACAAATCA	1	0
19060	CCGATTGGTAAAAACGAGTTG	GGCAAAAGGTCAGCTTGCTA	0	NA [a]
15550	TGAGGTGCCTTCTGAAACTG	CGTTAGCTGGTGCTGTGGT	0	NA [a]
12000	CCTGGTACAAGGCGGTTTC	CGGCTAACTGATACACCACCT	0	NA [a]
10280	TGCCATAGTGTGGATGGATG	GCTCACTTTCCAGTCTCCTGA	0	0
7800	CGTTGATGGTGGTGCTACTG	AACGTAAAGCTAACCACAAACAGA	0	0
6500	TTTGTTTGACGCCCCTTAAC	TTCAAACCAGCATCCACATT	0	NA [a]
5000	CGTGCCGGAGTAGATAGAGG	GAGAGGTTATAGATGGATCCGAT	0	NA [a]
BAC_BEG	AAGCGACAATGGAAGATTCG	CAGTGGAGTTCTCCCGGTTA	1	7
171R	GCGGGCGTTTCCAACTATTA	TGAATTGCACGGTAGTCCTG	7	NA [a]
Lp02_555	GTCAGAGCTCGCACCCTTC	CCCTTGGTGATCATTGCAC	67	51

Note: The populations have been screened for recombination using twelve markers, mapping within the *Z*-locus region. The fine-mapped *Z*-locus is defined by the region without any recombination events. In addition, the primer sequences of the polymorphic molecular markers used for the fine-mapping of the self-incompatibility locus *Z* are given.

[a] Markers were either monomorphic or did not amplify in DTZ

Supplementary Table 2: Primers used to investigate the expression pattern of *S*- and *Z*-locus genes using RT-qPCR.

	Gene name	Primer F	Primer R	Amplicon size	Primer efficiency
***S*-locus genes of interest**	*LpTIR1*	5'-CAATGTTGCTAGCCTGCTTC-3'	5'-AATGCTGGCAGGTAATCTGG-3'	176	1.79
	LpdsRNA	5'-AGAGTCGAAAAAGCCAGCAG-3'	5'-CTTGGTGGAGTTTGCATGTC-3'	180	1.76
	LpSNF2	5'-TGACCCATAAACACTGTCACG-3'	5'-CCCCATCATCTCATCCTAGTG-3'	184	1.81
	LpSDUF247-IA	5'-GAATGCGGTTCCAGAAAAGG-3'	5'-GCAGTGATGACATTCCCAAC-3'	181	1.77
	LpSDUF247-IB	5'-TCTGGGCTTGAGAAAGAAGC-3'	5'-CCTCTCGTCTACCATGTTTTCC-3'	180	1.82
	LpSDUF247-IIA	5'-AGAAACGAGCAAGGATGTGC-3'	5'-CTCGTGTATTTCGGCATGTG-3'	176	1.91
	LpSDUF247-IIB	5'-TGAGGACCATTCGGCTATTG-3'	5'-TAGCACCGTCCAATCTGTTG-3'	177	1.81
	LpsS-A	5'-CGTCATGGTTCTGATGGTTATG-3'	5'-GATGCCCAAATGGTTTTCC-3'	72	1.78
	LpsS-B	5'-CGGTTTCTCCGAATCTAAGG-3'	5'-CCTTGTACATGCAAGGTGGTC-3'	80	1.66
***Z*-locus genes of interest**	*LpZDUF247-IA*	5'-GGTGGCAATTGAGGAACTTG-3'	5'-CTCAAGAGTGCGCATTTCAC-3'	184	1.87
	LpZDUF247-IB	5'-GCGCAGAGTTGCACAATATC-3'	5'-CTCGAACGCAATAAGGTTCC-3'	175	1.79
	LpZDUF247-IIA	5'-GCGAGCAGTAGAATACCATCG-3'	5'-GGATCTGTTTGCTCAAGTGC-3'	183	1.77
	LpZDUF247B-IIB	5'-TGTCAGGCTAACCAAAGACG-3'	5'-GATGACCGCAGCAAATACAG-3'	176	1.80
	LpGK	5'-AACTGGTGCCTTCATCCTTC-3'	5'-TAATGATCCCAAGGCTGTCC-3'	179	1.77
	LpsZ-A	5'-TTACCCCCAACACTGAACAC-3'	5'-AACCAGGCTGTTTGGTATGG-3'	145	1.79
	LpsZ-B	5'-AACAAAGTGGTCGTCCTTCTG-3'	5'-GAGAAAGTGAATCCCCATGC-3'	97	1.78
**Reference genes**	*EF1-alpha*	5'-TTGACAAGCGTGTGATCGAG-3'	5'-TGACCAGGAGCATCAATGAC-3'	184	1.81
	eIF4A-2	5'-ATGCATGTGTTGGAGGAACC-3'	5'-CCTTGAAACCACGAGAAAGC-3'	183	1.80
	CPB20	5'-ACGCAGGAGGAGTTCGAG-3'	5'-GGTCTTGGTGTTCTTGTCGAG-3'	155	1.81
	eIF4A-1	5'-GAAAGACTGCCACCTTCTGC-3'	5'-CGAACAGATGTTCCACCAAC-3'	181	1.74

Note: The suffixes A and B in the gene name represent the two alleles of a specific gene. The displayed primer efficiencies were calculated using LinReg PCR 7.5 (Ramakers et al. 2003)

Supplementary Table 3: Quality and quantity assessment of the RNA samples used for RT-qPCR.

Tissue Type	Name	Weight [g]	Conc. Qbit [ng/µl]	RIN Value
**Anther**	Anther T_0 A	0.048	210	6.9
	Anther T_0 B	0.05	262	7.5
	Anther T_0 C	0.072	404	8.4
	Anther T_0 D	0.05	222	8.6
	Anther T_0 E	0.056	165	7
	Anther T_1 A	0.059	250	7.6
	Anther T_1 B	0.053	160	8.4
	Anther T_1 C	0.051	256	6
	Anther T_1 D	0.07	166	7.5
	Anther T_1 E	0.057	41	8.8
	Anther T_1 F	0.057	97	8.7
	Anther T_2 A	0.048	32	6.5
	Anther T_2 B	0.068	70	NA
	Anther T_2 C	0.074	50	7
	Anther T_2 D	0.053	60	8
	Anther T_2 E	0.066	52	8
	Anther T_2 F	0.066	54	8.4
**Stigma**	Stigma T_0 A	0.051	80	9.1
	Stigma T_0 B	0.055	62	9.3
	Stigma T_0 C	0.05	260	7.6
	Stigma T_0 D	0.055	78	9.1
	Stigma T_0 E	0.072	83	8.9
	Stigma T_1 A	0.04	50	7.6
	Stigma T_1 B	0.043	51	7.8
	Stigma T_1 C	0.045	42	5.6
	Stigma T_1 D	0.048	130	8.7
	Stigma T_1 E	0.044	74	9.5
	Stigma T_1 F	0.029	238	8.8
	Stigma T_2 A	0.047	122	9.1
	Stigma T_2 B	0.048	55	9.1
	Stigma T_2 C	0.065	120	9.1
	Stigma T_2 D	0.046	90	8.8
	Stigma T_2 E	0.051	129	9.1
**Leaf**	Leaf T_{NA} A	0.041	47	5.2
	Leaf T_{NA} B	0.054	31	4.5
	Leaf T_{NA} C	0.064	33	5
	Leaf T_{NA} D	0.056	117	5.5
	Leaf T_{NA} E	0.047	60	5.6
	Leaf T_{NA} F	0.046	51	5.3
